# Exercise training decreases the load and changes the content of circulating SDS-resistant protein aggregates in patients with heart failure with reduced ejection fraction

**DOI:** 10.1007/s11010-023-04884-z

**Published:** 2023-10-30

**Authors:** Marisol Gouveia, Cristine Schmidt, Priscilla Gois Basilio, Susana S. Aveiro, Pedro Domingues, Ke Xia, Wilfredo Colón, Rui Vitorino, Rita Ferreira, Mário Santos, Sandra I. Vieira, Fernando Ribeiro

**Affiliations:** 1 Department of Medical Sciences, iBiMED – Institute of Biomedicine, University of Aveiro, Building 30, Agras do Crasto - Campus Universitário de Santiago, Aveiro, 3810-193 Portugal; 2 Surgery and Physiology Department, Faculty of Medicine, University of Porto, Porto, Portugal; 3 Research Centre in Physical Activity, Health and Leisure, Faculty of Sport, University of Porto, Porto, Portugal; 4 Laboratory for Integrative and Translational Research in Population Health (ITR), Porto, Portugal; 5 Mass Spectrometry Centre, Department of Chemistry, LAQV REQUIMTE, University of Aveiro, Aveiro, Portugal; 6 GreenCoLab - Green Ocean Association, University of Algarve, Faro, Portugal; 7 Department of Chemistry and Chemical Biology, Rensselaer Polytechnic Institute, Troy, NY USA; 8 Centre for Biotechnology and Interdisciplinary Studies, Rensselaer Polytechnic Institute, Troy, NY USA; 9 Department of Chemistry, QOPNA & LAQV-REQUIMTE, University of Aveiro, Aveiro, Portugal; 10 Serviço de Cardiologia, Hospital Santo António, Centro Hospitalar Universitário do Porto, Porto, Portugal; 11 Instituto de Ciências Biomédicas Abel Salazar, UMIB, University of Porto, Porto, Portugal; 12 School of Health Sciences, iBiMED – Institute of Biomedicine, University of Aveiro, Aveiro, Portugal

**Keywords:** HFrEF, Exercise training, Protein aggregates, Proteomics

## Abstract

**Background:**

Heart failure (HF) often disrupts the protein quality control (PQC) system leading to protein aggregate accumulation. Evidence from tissue biopsies showed that exercise restores PQC system in HF; however, little is known about its effects on plasma proteostasis.

**Aim:**

To determine the effects of exercise training on the load and composition of plasma SDS-resistant protein aggregates (SRA) in patients with HF with reduced ejection fraction (HFrEF).

**Methods:**

Eighteen patients with HFrEF (age: 63.4 ± 6.5 years; LVEF: 33.4 ± 11.6%) participated in a 12-week combined (aerobic plus resistance) exercise program (60 min/session, twice per week). The load and content of circulating SRA were assessed using D2D SDS-PAGE and mass spectrometry. Cardiorespiratory fitness, quality of life, and circulating levels of high-sensitive C-reactive protein, N-terminal pro-B-type natriuretic peptide (NT-proBNP), haptoglobin and ficolin-3, were also evaluated at baseline and after the exercise program.

**Results:**

The exercise program decreased the plasma SRA load (% SRA/total protein: 38.0 ± 8.9 to 36.1 ± 9.7%, p = 0.018; % SRA/soluble fraction: 64.3 ± 27.1 to 59.8 ± 27.7%, p = 0.003). Plasma SRA of HFrEF patients comprised 31 proteins, with α-2-macroglobulin and haptoglobin as the most abundant ones. The exercise training significantly increased haptoglobin plasma levels (1.03 ± 0.40 to 1.11 ± 0.46, p = 0.031), while decreasing its abundance in SRA (1.83 ± 0.54 × 10^11^ to 1.51 ± 0.59 × 10^11^, p = 0.049). Cardiorespiratory fitness [16.4(5.9) to 19.0(5.2) ml/kg/min, p = 0.002], quality of life, and circulating NT-proBNP [720.0(850.0) to 587.0(847.3) pg/mL, p = 0.048] levels, also improved after the exercise program.

**Conclusion:**

Exercise training reduced the plasma SRA load and enhanced PQC, potentially via haptoglobin-mediated action, while improving cardiorespiratory fitness and quality of life of patients with HFrEF.

**Supplementary Information:**

The online version contains supplementary material available at 10.1007/s11010-023-04884-z.

## Introduction

Heart failure (HF) is a complex syndrome with a dramatic negative impact on the quality of life and functional capacity of patients [1, 2]. Despite the remarkable advances in cardiovascular medicine, patients with HF still show poor prognosis with high readmission and mortality rates [3–5]. At the cellular level, a wide diversity of factors has been associated with maladaptive cardiac remodelling and HF development [6, 7], such as neurohormonal hyperactivation [8, 9], oxidative stress [10], and impairment of the protein quality control (PQC) system [11–13]. Myocardium tissue continuously suffers remodelling to ensure its optimal mechanical function, in which the protein homeostasis (proteostasis) is conserved by the constant production and degradation of the cardiac proteins [14]. These processes are controlled by a complex quality control system that involves, for instance, protein-degradation machinery (e.g. ubiquitin-proteasome system [UPS] and autophagy), and the endoplasmic reticulum unfolded protein response (UPR) that reduces unfolded protein accumulation by e.g., inducing molecular chaperone expression to assist the protein folding [15, 16]. However, prolonged or severe cardiac stress promoted by, e.g., hypoxia [17], oxidative stress [18], and high blood pressure [19], contributes to overload or failure of the PQC system and increased protein misfolding and aggregation [20]. An impaired PQC system has been linked to ageing [21], atherosclerosis [22, 23], hypertension [24, 25], and HF [26–29]. Indeed, extracellular deposition of amyloid aggregates in the myocardium is frequent in older adults and has been implicated in HF development [30, 31]. Recently, we found that patients with HF with preserved ejection fraction (HFpEF) displayed higher levels of plasma SDS-resistant protein aggregates (SRA) than age-matched individuals, suggesting a compromised PQC machinery to counteract SRA burden [32]. SRA are hyper-stable structures, resistant to sodium dodecyl sulfate (SDS)-induced denaturation, that may accumulate in the organism due to their ability to overcome protein degradation systems. The accumulation of hyper-stable aggregates can lead to the sequestration and dysfunction of important interacting partners, as well as to the disruption of organ architecture and function [32–35]. Also, Gene Ontology (GO) enrichment analysis indicated that the proteomic profile of these SRA was composed of extracellular proteins and most of them possess endopeptidase inhibitor activity and participate in several regulatory biological processes [32]. The GO annotation enrichment analysis used the GO system to classify gene products according to the biological process, molecular function, and cellular component [36].

Exercise training is highly recommended as adjuvant therapy to HF management since it improves exercise tolerance and quality of life while reducing HF-related hospitalizations [2, 37]. Moreover, evidence gathered mostly from animal studies indicates that exercise training restores PQC systems and/or reduces the burden of protein aggregates in several cardiovascular disease models [38–41]. For instance, in a post-myocardial infarction-induced HF animal model, 8-week aerobic exercise increased cardiac function and improved proteostasis by restoring the PQC degradation systems [42, 43], and attenuated endoplasmic reticulum stress and UPR activation [44]. Aerobic exercise also reduced oxidative stress and UPS overactivation in skeletal muscles, reducing muscle atrophy and exercise intolerance, in sympathetic hyperactivity-induced HF mice [45]. The same study showed that exercise-trained HF patients showed improved aerobic capacity and restored proteasomal activity in skeletal muscle [45]. However, animal findings may not translate into clinical ones [46, 47] or biopsies may be necessary to evaluate the impact of a therapeutic intervention on PQC. It is thus crucial to assess the impact of exercise training on the proteostasis of easily assessed biological fluids, such as plasma samples. Blood samples, collected via minimally invasive methods, may remove the practical obstacles that have been holding back the study of PQC systems and protein aggregation in routine clinical practice. Thus, this work aimed to study the effect of a 12-week exercise training program on the load and composition of protein aggregates in the plasma of patients with HF. We also aimed to determine the effects of the exercise training program on clinical parameters such as cardiorespiratory fitness, quality of life, and circulating biomarkers.

## Methods

### Study population

Eighteen patients with HFrEF were enrolled in this prospective study. The recruitment was performed at the Unit of Cardiac Rehabilitation of the Cardiology Department of Centro Hospitalar Universitário do Porto - Hospital de Santo António, Porto, Portugal. The inclusion criteria included patients aged ≥ 18 years with a diagnosis of HFrEF according to the criteria of the European Society of Cardiology [2], clinical stability and optimal medical treatment for ≥ 6 weeks, and patients able to follow the exercise prescription. Exclusion criteria: patients who have undertaken cardiac rehabilitation within the past 12 months; implantable cardioverter-defibrillator (ICD), cardiac resynchronization therapy (CRT), or combined CRT/ICD device implanted in the last 6 weeks; myocardial infarction in the last 3 months; ischaemia signs during cardiopulmonary exercise testing; symptomatic and/or exercise-induced cardiac arrhythmia or conduction disturbances; currently pregnant or intend to become pregnant in the next year; inability to exercise or conditions that may interfere with exercise intervention; expectation of receiving a cardiac transplant in the next 6 months; participation in another clinical trial; patients who are unable to understand the study information or complete the questionnaires. The study was approved by the hospital Ethics Committee [2019/123(103-DEFI/107-CE)]. Participants provided written informed consent and all procedures followed the Declaration of Helsinki.

### Procedures

#### Clinical variables

Clinical files were consulted to obtain the patients’ demographic and clinical data, medical history, medication, and New York Heart Association (NYHA) functional class. Data were confirmed with the patient and/or the clinician. A standard wall-mounted stadiometer and scale were used to assess height and weight, respectively. Blood pressure (BP) was assessed according to the ESH/ESC guidelines [48]. Left ventricular ejection fraction (LVEF) was assessed by transthoracic echocardiography with a cardiovascular ultrasound (Vivid E95®, GE Healthcare, Chicago, IL, USA) following the ESC guidelines [49].

At baseline and after the exercise program, N-terminal pro-B-type natriuretic peptide (NT-proBNP) and high-sensitive C-reactive protein (hsCRP) were evaluated. Cardiorespiratory fitness, measured as peak oxygen uptake (VO_2_ peak), was assessed using an ergospirometry device during a maximal or symptom-limited treadmill exercise test using the modified Naughton or Bruce protocol. Functional exercise capacity was determined with the 6-minute walk test (6 MWT) [50]. Health-related quality of life was assessed with the Minnesota Living with Heart Failure Questionnaire (MLHFQ) [51]. The MLHFQ comprises 21 items rated on a six-point Likert scale from 0 (‘no’) to 5 (‘very much’), providing scores for two dimensions, physical and emotional, and a total score.

#### Exercise training intervention

HF patients participated in a 12-week combined (aerobic plus resistance) exercise program with 2 training sessions per week, for a total of 24 sessions. Each training session was divided into 4 sections/parts: (i) 5–10 min of warm-up with calisthenics and stretching exercise; (ii) 30 min of aerobic exercise on a treadmill or cycle ergometer, or walking training at 60-80% of VO_2_ peak (11–14 Borg´s scale); (iii) 25 min of resistance exercises with a combination of calisthenics exercises and an elastic band exercise; 2 sets of 12–15 repetitions of 10 exercises (squat, leg curl, leg abduction, leg adduction, standing calf raise, bench press sitting, seated row, biceps, triceps, lateral raises); and (iv) 5 min of cool-down with stretching exercises.

#### Blood sampling and protein quantification

Peripheral venous blood was drawn into ethylenediaminetetraacetic acid (EDTA) tubes at baseline and after the 12-week intervention (48 h after the last training session). Plasma samples were separated by centrifugation (15 min at 2000 g at 4 ºC) and stored at -80 ºC. Protein quantification was carried out with the bicinchoninic acid (BCA) protein assay kit (Pierce®, Thermo Scientific, Massachusetts, USA).

#### Analysis of SDS-resistant protein aggregates

A detailed description of the diagonal two-dimensional (D2D) SDS-PAGE assay, developed by Xia et al. [33], was applied to SRA analysis as previously described [32]. The D2D SDS-PAGE assay was employed in the pre- and post-exercise samples of 5 randomly selected participants. In brief, for the isolation of SRA by D2D SDS-PAGE, plasma samples were depleted from the two most abundant plasma proteins (albumin and IgG). For the first dimension, protein sample (150 µg) was incubated with 1 x SDS sample buffer and electrophoresed in 12% SDS-PAGE gel. Next, the gel lane was cut out and boiled in SDS equilibration buffer with DTT, followed by alkylation with iodoacetamide. Then, the gel lane was placed on top of a 12% polyacrylamide gel and electrophoresis was performed at 55 mA/gel. Coomassie-stained gels were digitalized in the GS-800 calibrated densitometer (Bio-Rad, California, USA) and protein bands quantification was performed with ImageLab software (Bio-Rad) by a blinded examiner.

*Spots digestion and protein identification by liquid chromatography-mass spectrometry*.

SRA spots were washed, reduced, alkylated, and digested with trypsin overnight. Then, peptides were extracted and sent for mass spectrometry analysis. A detailed description of the mass spectrometry analysis can be found here [32]. In 4 samples of each assessment moment (baseline and post-exercise), SRA spots #1–6 were analysed as a pool; in 1 sample per moment, each SRA spot was analysed individually. The separation of peptides was performed on a Q Exactive Orbitrap through the EASY-spray nano ESI source (Thermo Fisher Scientific, Bremen, Germany) coupled to an Ultimate 3000 (Dionex, Sunnyvale, CA) HPLC system. Spectra were processed and database searching was performed using MS Amanda, Sequest HT, and percolator algorithms embedded in Proteome Discoverer (version 2.2, Thermo Scientific). All searches were performed against the UniProtKB/Swiss-Prot human proteome sequence database (version of February 2018). The following database search parameters were applied: carbamidomethylation of cysteine (C) was defined as a static modification, and oxidation of methionine (M) and acetylation of N-terminal protein (N-Terminus) were set as dynamic modifications and up to two trypsin missed cleavages were considered. Only proteins found at least twice on each set of samples (baseline and post-exercise) were considered. The posttranslational modifications oxidation and dioxidation were searched through the analysis of mass shifts of + 15.995 and + 31.990 Da, respectively. The mass spectrometry proteomics data have been deposited to the ProteomeXchange Consortium via the PRIDE [52] partner repository with the dataset identifier PXD045161.

*Bioinformatics analysis*.

The protein-protein interaction (PPI) network and the functional enrichment analysis were performed with the search tool for the retrieval of interacting genes/proteins (STRING) version 11.0 (https://string-db.org) [53].

#### Immunoblot analysis

Protein samples (50 µg) in SDS sample buffer were separated in 10% SDS-PAGE gel, transferred to nitrocellulose membranes, and blocked with 5% non-fat dry milk in TBS-T. The membranes were probed overnight at 4ºC with antibodies for haptoglobin (#ab131236, Abcam, United Kingdom, 1:10000) and ficolin-3 (#AF-2367, R&D systems, United Kingdom, 1:10000). After incubation with the proper horseradish-peroxidase (HRP)-conjugated secondary antibody, membranes were visualized using the enhanced chemiluminescence method (Amersham ECL Prime WB detection kit, RPN2236) and ChemiDocTM image acquisition system (Bio-Rad, USA). Densitometry values of the proteins’ signals were standardized to a reference sample, allowing for comparing samples among different blots. The resulting optical density signal was normalized to Ponceau S staining, a loading control.

#### Data analysis

SPSS version 27.0 (SPSS IBM Corporation, Chicago, IL, USA) was used to carry out all statistical analyses. The normality of data distribution was evaluated with the Shapiro–Wilk test, histogram analysis, and checked for kurtosis and skewness. Descriptive statistics (mean ± SD or median (IQR), counts, percentages, and ratios) were computed for sample characterization. Paired t-tests or Wilcoxon signed-rank tests were performed for comparisons from baseline to the end of the exercise. The level of significance was set as a P value less than 0.05.

## Results

### Baseline characteristics and clinical data of the participants

The baseline characteristics of the participants are displayed in Table 1. The mean age of the patients was 63.4 ± 6.5 years old, mostly men (78%), and most of them were in NYHA functional class II (78%).

**Table 1:** Demographics and clinical characteristics of the HFrEF patients at baseline

Characteristics	N = 18
**Age (years)**	63.4 ± 6.5
**Sex (Female/Male)**	4/14
**LVEF (%)**	33.4 ± 11.6
**Ischaemic aetiology, n (%)**	9 (50)
**NYHA functional class, n (%)**	
**I**	3 (17)
**II**	14 (78)
**III**	1 (6)
**Medical history, n (%)**	
**Overweight/ Obesity**	9 (50)
**Currently smoking**	5 (28)
**Hypertension**	7 (39)
**Diabetes**	8 (44)
**Dyslipidaemia**	15 (83)
**Atrial fibrillation**	4 (22)
**CAD**	6 (33)
**PAD**	3 (17)
**OSA**	1 (6)
**Previous stroke**	2 (11)
**Previous MI**	6 (33)
**Kidney disease**	4 (22)
**Medication/devices, n (%)**	
**ACE-I and/or ARB**	11 (61)
**β-blocker**	18 (100)
**Loop diuretic**	12 (67)
**MRA**	13 (72)
**Digoxin**	1 (6)
**Statin**	15 (83)
**Anticoagulant**	6 (33)
**Antiaggregant/antiplatelet**	9 (50)
**Antiarrhythmic**	2 (11)
**Sacubitril/Valsartan**	5 (28)
**Antidiabetic**	7 (39)
**SGLT2i**	3 (17)
**CPAP**	2 (11)
**ICD**	5 (28)
**CRT**	4 (22)

Data are mean ± standard deviation or number (%). LVEF, left ventricular ejection fraction; NYHA, New York Heart Association; CAD, coronary artery disease; PAD, peripheral artery disease; OSA, obstructive sleep apnoea; MI, myocardial infarction; ACE-I, angiotensin-converting enzyme inhibitor; ARB, angiotensin receptor blocker; MRA, mineralocorticoid receptor antagonist; SGLT2i, sodium-glucose co-transporter 2 inhibitor; CPAP, continuous positive airway pressure therapy; ICD, implantable cardioverter-defibrillator; and CRT, cardiac resynchronization therapy

### Effect of exercise training on clinical variables

After the exercise program, NT-proBNP levels [-13.5 (33.1)%, p = 0.048], the 6MWT performance (10.6 ± 7.7%, p < 0.001), and the VO_2peak_ [8.4 (19.4)%, p = 0.002] improved significantly (Table 2). Health-related quality of life also improved [-50.9 (79.1)%, p = 0.004], mostly due to an improvement in the physical dimension of the MLHFQ (Table 2).

**Table 2:** Changes in clinical variables from baseline to the end of the 12-week exercise training program

Characteristics	Baseline	12 weeks	Change from baseline	*P* value
**BMI (kg/m** ^**2**^ **)**	27.3 ± 3.9	27.0 ± 4.1	0.3 ± 0.8	0.053
**SBP (mmHg)**	117.3 ± 17.6	115.3 ± 14.8	-2.0 ± 15.0	0.290
**DBP (mmHg)**	67.6 ± 12.0	68.6 ± 11.8	1.0 ± 13.8	0.813
**Heart rate (bpm)**	67.9 ± 11.5	70.5 ± 10.0	2.6 ± 11.3	0.175
NT-proBNP (pg/mL)*	720.0 (850.0)	587.0 (847.3)	-57.0 (266.8)	0.048
hsCRP (mg/mL)* ^**#**^	1.5 (1.4)	1.2 (1.5)	-0.1 (1.6)	0.532
**6MWT (m)**	461.2 ± 64.4	508.2 ± 65.5	47.1 ± 33.4	< 0.001
**VO**_**2peak**_ (mL/min/Kg)*	16.4 (5.9)	19.0 (5.2)	1.9 (2.6)	0.002
**MLHFQ (points)**				
Physical dimension	12.0 (16.0)	4.0 (10.5)	-4.5 (8.0)	0.009
Emotional dimension	4.0 (4.0)	3.0 (8.5)	0.0 (3.0)	0.282
Total	25.0 (26.5)	13 (30.5)	-9.0 (13.5)	0.004

Data are mean ± standard deviation or *median and interquartile range (IQR). BMI, body mass index; SBP, systolic blood pressure; DBP, diastolic blood pressure; NT-proBNP, N-terminal pro-B-type natriuretic peptide; hsCRP, high-sensitive C-reactive protein; 6MWT, six-minute walk distance test; VO_2peak_, peak oxygen uptake, and MLHFQ, Minnesota Living with Heart Failure Questionnaire. ^#^ n = 15

### Effect of exercise training on the load of SDS-insoluble protein aggregates

**Fig. 1:**
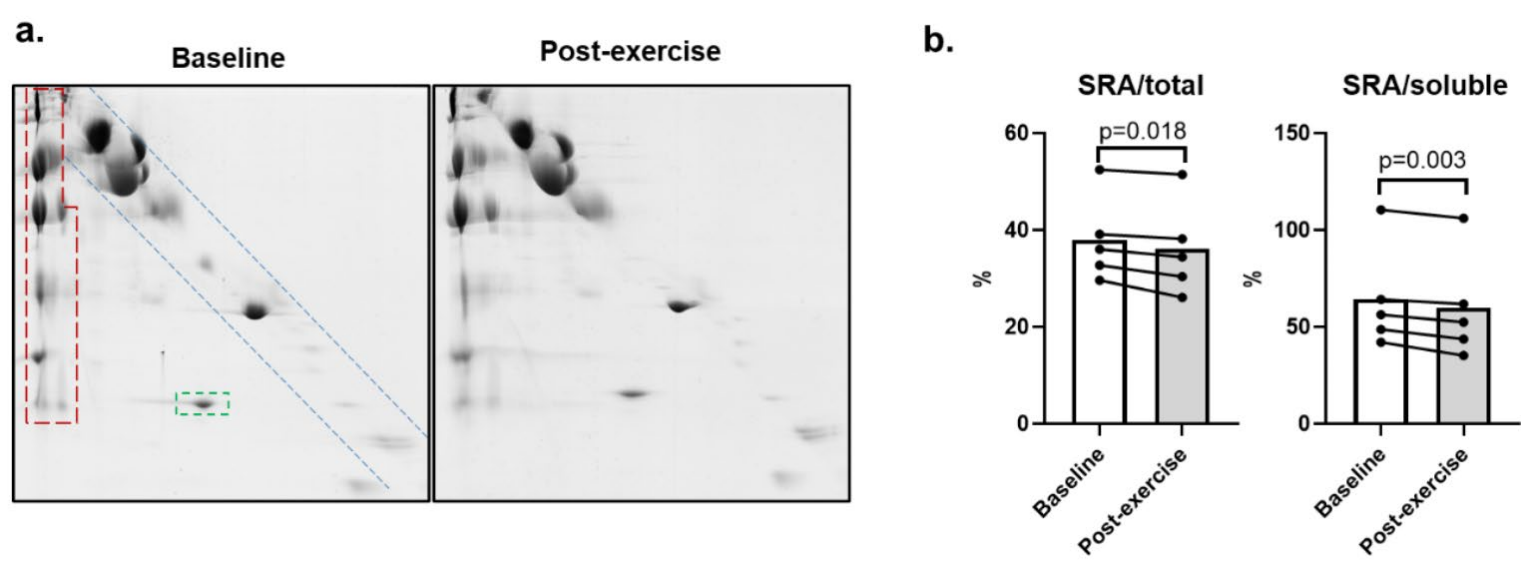
Analysis of plasma samples of HFrEF patients by D2D SDS-PAGE. **(a)** Representative D2D SDS-PAGE gels of plasma samples of patients with HFrEF before (baseline) and after 12 weeks of exercise training (post-exercise). D2D SDS-PAGE gel displays: in the blue box, the usual electrophoretic diagonal pattern of non-SDS-resistant, soluble, proteins, that migrate equal distances in both dimensions; in the green box, SDS-resistant proteins that are located below the diagonal soluble proteins pattern, since they migrate less in the first electrophoretic run; in the red box, SDS-resistant aggregates (SRA), that do not resolve in the first electrophoretic dimension (remain at the interface between the stacking and the resolving gels), but resolve in the second dimension (run vertically), after boiling the samples with SDS and DTT, which dissociate SRA complexes into their subunits. **(b)** Percentage of relative abundance between SRA and all proteins present in the gel (‘SRA/total’) or between SRA and the soluble protein fraction (‘SRA/soluble’) in the D2D gels, before (‘Baseline’) and after (‘Post-exercise’) the exercise training program. Mean data are presented in graphs (n = 5 per condition)

SDS-insoluble protein aggregates (SRA) were resolved by D2D gels. Representative images are shown in Fig. 1a (all gels are presented in Supplementary Fig. 1). Overall, baseline and post-exercise program plasma samples presented a similar 2D migration pattern of protein aggregates (Supplementary Fig. 1). After the exercise training, the relative levels of SRA expressed as a percentage of the whole protein content decreased significantly (SRA/total: 38.0 ± 8.9 to 36.1 ± 9.7%, p = 0.018). Likewise, SRA levels expressed as a percentage of the soluble fraction (SRA/soluble) decreased from 64.3 ± 27.1 to 59.8 ± 27.7% (p = 0.003) (Fig. 1b). The relative abundance of SDS-resistant proteins (Fig. 1a, green box) also decreased significantly, from 1.32 ± 0.48 to 1.12 ± 0.39% (p = 0.012).

### Content analysis of SDS-insoluble protein aggregates in plasma

**Fig. 2:**
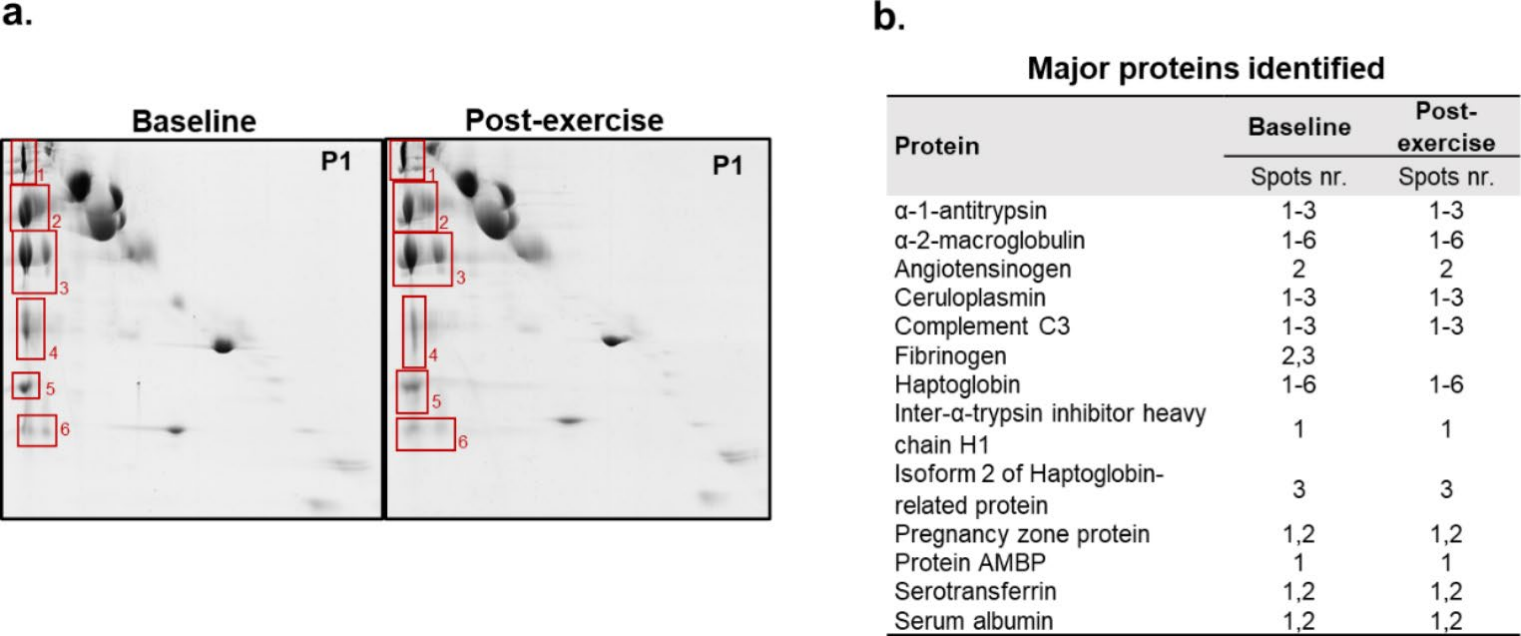
D2D gels of pre- and post-exercise plasma samples from a patient with HFrEF. **a**. Selected protein spots analysed by mass spectrometry are numbered. **b**. Distribution by spots 1–6 of the main proteins identified in at least 4 samples at both assessment moments, baseline and post-exercise intervention

For identification of SRA protein constituents by mass spectrometry, the SRA spots were evaluated individually on two representative D2D gels from one patient with HFrEF (pre- and post-exercise training gels, Fig. 2a). Further, in the baseline and post-exercise training samples of the other 4 HFrEF patients, the SRA spots of each gel were analysed as a pool (Supplementary Fig. 1). A detailed list of all proteins identified in SRA spots is provided in Supplementary Table 1. A total of 31 different proteins were identified and were common to baseline and after the 12-week exercise training program. Of note, some immunoglobulins were also detected in this study (Supplementary Table 2). The main proteins identified before and after exercise training are provided in Fig. 2b.

**Fig. 3:**
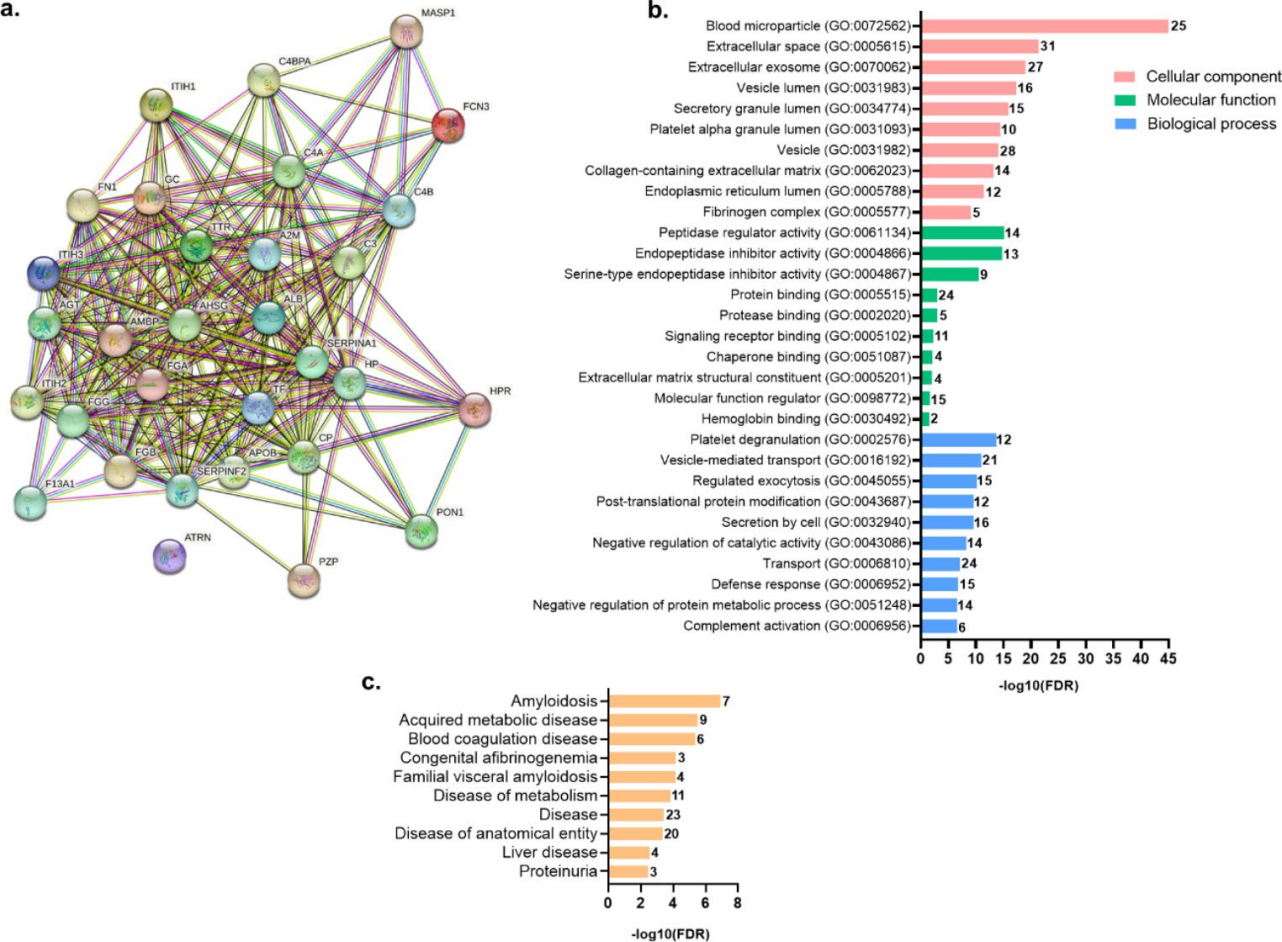
STRING analysis of the protein components of SDS-resistant aggregates identified in common between pre- and post-exercise plasma samples of HFrEF patients. **a.** PPI network where each node represents a protein, and the edges represent their interactions. The correspondence of gene ID to protein name is presented in Supplementary Table 1. **b.** Scheme with the top 10 most significantly enriched GO terms regarding molecular component, molecular function, and biological process. **c**. Top disease enrichment analysis. The bars represent the -log10 of the false discovery rate (FDR) for the corresponding classification, and the number in the bar represents the number of proteins enriched in each term

To gain valuable biological insight into the identified proteins in SDS-resistant aggregates, the STRING database was employed for protein-protein interaction (PPI) network analysis and to perform GO annotation enrichment analysis. The PPI network retrieved by STRING revealed a network of dense interactions between the identified SRA proteins (Fig. 3a).

Relatively to these proteins’ properties, Fig. 3b shows the top 10 most statistically significant GO enrichment terms. All the proteins identified in SRA were found in the extracellular space, and most of them were also associated with the GO cellular component terms “blood microparticle”, “extracellular exosome”, and “vesicle” (Fig. 3b, in salmon). Concerning the enriched GO molecular functions, most proteins were related to the regulation of peptidase activities, and binding to proteins, including proteases and chaperones (Fig. 3b, in green). This chaperone binding function was attributed to four of the identified aggregated proteins, namely ceruloplasmin, albumin, fibrinogen, and fibronectin 1. Regarding the top GO biological processes (Fig. 3b, in blue), most of the identified SRA were involved in platelet degranulation, post-translational modifications, vesicle-mediated transport, and regulation of exocytosis, besides defence response. Finally, enrichment analysis of diseases to which the identified proteins have been related to retrieved amyloidosis and metabolic diseases as highly enriched (Fig. 3c). SRA proteins transthyretin, complement C3, albumin, fibrinogen, α-2-macroglobulin, fibronectin 1, and α-1-antitrypsin have been related to amyloidosis.

Quantitative analyses of the average abundances of the identified proteins (Supplementary Tables 1 and 3), revealed that the main components of SRA in plasma samples of HFrEF patients, were α-2-macroglobulin and haptoglobin, corresponding to > 95% of the SRA protein content (Supplementary Fig. 2). These were also the two SRA proteins more heavily oxidized, a post-translational modification associated with misfolding and aggregation, that was also retrieved by the mass spectrometry analysis (Supplementary Tables 4 to 7).

### Effect of exercise training on SRA proteins abundances and haptoglobin levels

The relative abundances of each SRA protein at baseline and after the exercise training are presented in Supplementary Tables 1 and 3. Only SRA proteins identified in baseline and post-exercise paired samples of at least two patients were considered. Exercise training significantly decreased the amount of haptoglobin in SRA, from 1.83 × 10^11^ ± 0.54 × 10^11^ at baseline to 1.51 × 10^11^ ± 0.59 × 10^11^ (p = 0.049) after the exercise training program, corresponding to an average decrease of 19.8 ± 16.6% (Supplementary Table 1).

**Fig. 4:**
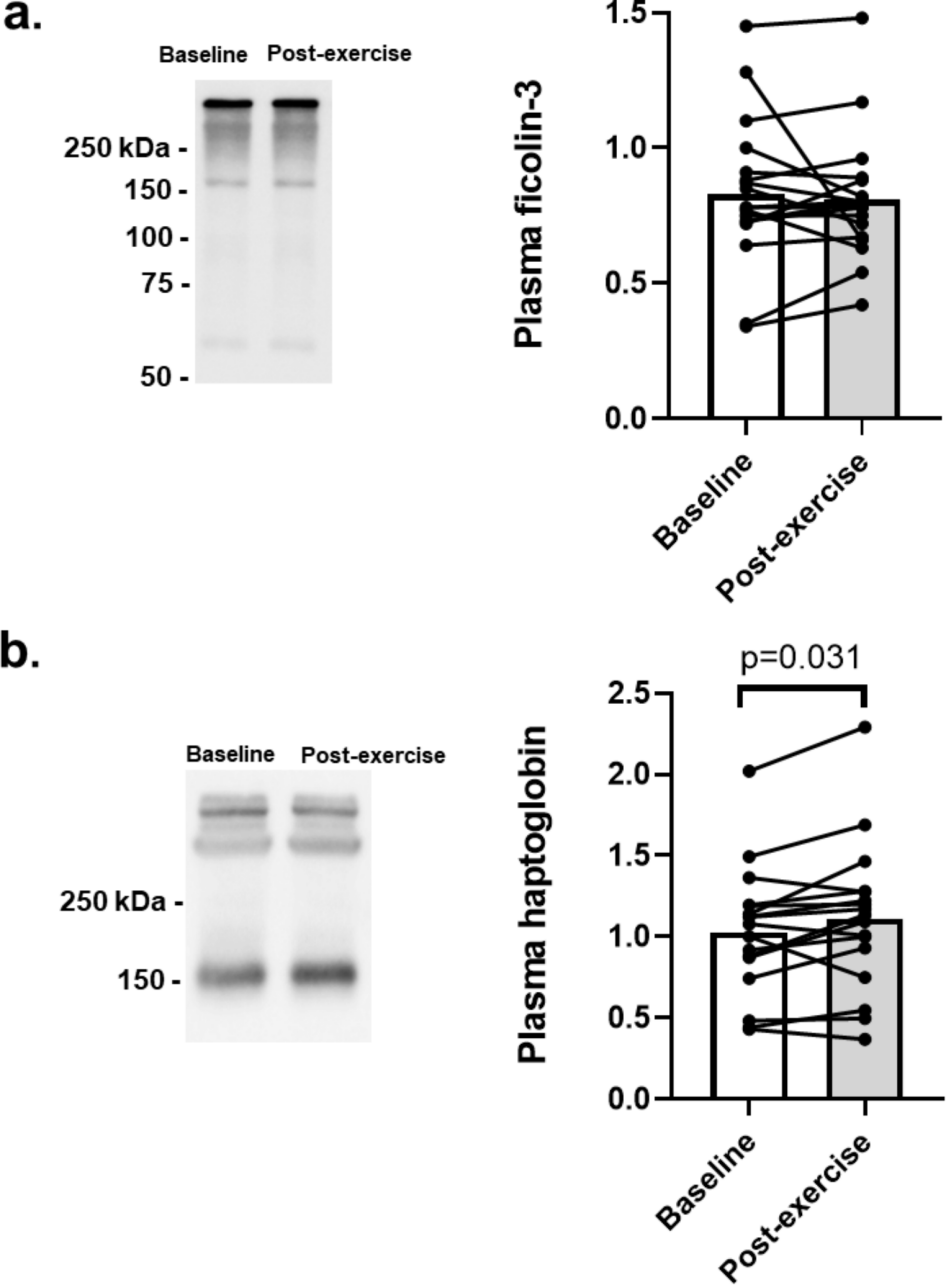
Effect of 12 weeks of exercise training on ficolin-3 and haptoglobin levels in the plasma from patients with HFrEF. Representative immunoblots and graphic analyses of **(a)** ficolin-3 (n = 18) and **(b)** haptoglobin (n = 17) levels in 1D SDS-PAGE gels, run in non-DTT reducing conditions. Haptoglobin is present in plasma as different types of polymeric forms with high molecular weight, or as tetrameric proteins composed of two α-chains (α1 and/or α2) and two conserved β-chains; the anti-haptoglobin antibody used detects its β-chain, and thus all the native haptoglobin forms

The effect of exercise training on the plasma levels of haptoglobin and of another SRA protein, ficolin-3, was evaluated by immunoblotting under non-DTT reducing conditions (Fig. 4). Patients presented similar ficolin-3 levels after exercise training (0.83 ± 0.27 to 0.81 ± 0.23, p = 0.622; Fig. 4a). As shown in Fig. 4b, the haptoglobin levels increased significantly after the exercise training program (1.03 ± 0.40 to 1.11 ± 0.46, p = 0.031).

## Discussion

This study assessed the effects of a 12-week exercise training program on the extracellular proteostasis of patients with HFrEF, particularly the SRA insoluble protein aggregates. Exercise training significantly decreased both the total amount of plasma SRA and the amount of haptoglobin in SRA, while increasing the plasma haptoglobin levels (hence, in its more soluble form). Given the chaperone activity of haptoglobin, these data suggest a better extracellular proteostasis. Exercise training also improved NT-proBNP levels, cardiorespiratory fitness, functional exercise capacity (walking distance), and health-related quality of life of HFrEF patients.

Exercise training has been reported to induce a myriad of positive effects in multiple organ systems, improving inflammation, oxidative stress, sensitivity to insulin, glucose control, cardiac output, neurogenesis, and neuroplasticity [54–57]. In this study, we observed several benefits with clinical impact as a result of a 12-week exercise training program in patients with HFrEF. Patients showed an improvement in VO_2_ peak of 8.4(19.4)%, clinically relevant since every 6% increase in VO_2_ peak over 3 months has been associated with an 8% lower risk for cardiovascular mortality or HF hospitalization [58]. Furthermore, patients increased the 6MWT distance by 47.1 ± 33.4 m, and 30–50 m are considered to be the minimum clinically important difference in 6MWT, linked to an improvement of NYHA functional class [59]. These functional improvements are reflected in a better health-related quality of life, indicated by a 9-point improvement in the MLHFQ, with a decrease of ≥ 5 in the MLHFQ score already considered to be clinically meaningful [60]. Together with the NT-proBNP reduction, these improvements are strong indications of the benefits of exercise intervention among this patient population.

HF is multifactorial, and the cardiotoxicity of misfolded and aggregated proteins constitutes one of the mechanisms associated with HF development [11, 13, 20, 26]. Exercise training has been shown to counteract the proteotoxicity of misfolded proteins by the re-establishment of PQC systems [38, 43, 61–63]. Likewise, in previous studies using animal models or tissue collected during a biopsy, we also observe significant changes in the protein aggregation after exercise training, using a plasma sample. In a mice model of desmin-related cardiomyopathy, a 22-week exercise intervention decreased pre-amyloid oligomers formation, decelerated HF progression, and increased lifespan [64]. A study in older mice showed that a 12-week treadmill running program improved autophagic flux, clearance of ubiquitinated proteins, and protein aggregates [61]. Additionally, in both animal models and patients with HF, exercise training has been shown to prevent or reverse skeletal muscle wasting by reducing excessive protein degradation, which was associated with the restoration of redox balance and reduction of UPS hyperactivity [45, 63, 65]. Indeed, after 3 weeks of treadmill walking, HFrEF patients exhibited a re-establishment of skeletal muscle proteasomal activation to normal levels, which was accompanied by improvement in aerobic capacity [45]. Also, in the LEICA study, after 4-weeks of endurance training, HF patients showed reduced E3 ligase MuRF-1 levels, an enzyme that targets proteins to proteasomal degradation, and lower ubiquitinated protein levels in skeletal muscle biopsies [63].

In our study, we observed a decrease in plasma SRA load after a 12-week exercise training program among HFrEF patients. MS analysis revealed that these SRA are composed of at least 31 proteins, with α-2-macroglobulin and haptoglobin as the most abundant ones. These proteins are extracellular chaperones that inhibit aggregation of misfolded proteins in the extracellular environment [66, 67]. Also, both α-2-macroglobulin and haptoglobin were highly oxidized, a post-translational modification associated with protein misfolding and aggregation [68, 69]. Functional analyses characterized SRA proteins as mainly extracellularly located and with known functions in the regulation of exocytosis, defence response, and post-translational modifications, all processes already associated with protein aggregation. The presence of chaperones in extracellular insoluble protein aggregates has been reported in proteinopathies like amyloidotic cardiomyopathy [70], Alzheimer’s disease [71] and TTR amyloidosis [72], and related to failed attempts to keep misfolded proteins in a soluble state, under conditions of a large excess of aggregation-prone proteins [70, 72, 73].

Regarding the impact of exercise training on the SRA content, although most of the 31 SRA proteins were present both at baseline and after the exercise intervention, the exercise program significantly decreased the haptoglobin SRA levels. Given haptoglobin’s high abundance in SRA, this decrease is probably the main contributor to the observed exercise-induced decrease in SRA load. Haptoglobin has been proposed to act as a clusterin-like chaperone, limiting the toxicity of protein aggregates [74–76], and functioning as an anti-inflammatory protein [77]. Interestingly, the exercise training simultaneously decreased haptoglobin in SRA while increasing its total plasma levels. Taken together, these results suggest improved proteostasis, which includes higher levels of this chaperone-like protein, and more successful activity of haptoglobin on misfolded protein complexes. Increased haptoglobin levels may also aid in decreasing oxidative stress [78].

Limitations of this study include the small sample size that, given the phenotypic diversity and complexity of HF, limits the generalisability of these results. Future larger randomized controlled trials are thus required to validate our preliminary findings and to correlate improvements in extracellular proteostasis with clinical variables. More research on the value of other modes and frequencies of exercise on proteostasis is also needed. Further, the D2D SDS-PAGE assay is a complex and time-consuming technique in which each sample is analysed one by one by two sequential electrophoreses; for this reason, it was only applied to 5 (randomly selected) patients.

## Conclusion

A 12-week exercise training intervention improves extracellular proteostasis in patients with HFrEF, besides increasing relevant clinical variables. Exercise training reduced plasma SRA load, which contains proteins associated with amyloidosis and chaperones, among others. Particularly, it significantly decreased the amount of aggregated haptoglobin in SRA, while increasing its total circulating levels. Given this protein’s anti-oxidative and chaperone functions, the increase in more soluble haptoglobin form suggests an improved extracellular proteostasis control.

## Electronic supplementary material

Below is the link to the electronic supplementary material.

Supplementary Material 1
