# Supplementary material for: Exercise training decreases the load and changes the content of circulating SDS-resistant protein aggregates in patients with heart failure with reduced ejection fraction
Source: Mol Cell Biochem. 2023 Oct 30;479(10):2711–22. doi: 10.1007/s11010-023-04884-z (PMC11455743; doi:10.1007/s11010-023-04884-z)
Supplement: Supplementary file 1 — Supplementary Material 1 [file 11010_2023_4884_MOESM1_ESM.docx]

**Exercise training decreases the load and changes the content of circulating SDS-resistant protein aggregates in patients with heart failure with reduced ejection fraction**

Marisol Gouveia^1^, Cristine Schmidt^2,3,4^, Priscilla Gois Basilio^3^, Susana S. Aveiro^5,6^, Pedro Domingues^5^, Ke Xia^7,8^, Wilfredo Colón^7,8^, Rui Vitorino^1,2^, Rita Ferreira^9^, Mário Santos*^4,10,11^, Sandra Vieira^1*^, Fernando Ribeiro^12*^

^1^ iBiMED – Institute of Biomedicine, Department of Medical Sciences, University of Aveiro, Aveiro, Portugal

^2^ Surgery and Physiology Department, Faculty of Medicine, University of Porto, Porto, Portugal

^3^ Research Centre in Physical Activity, Health and Leisure, Faculty of Sport, University of Porto, Porto, Portugal

^4^ Laboratory for Integrative and Translational Research in Population Health (ITR), Porto, Portugal

^5^ Mass Spectrometry Centre, LAQV REQUIMTE, Department of Chemistry, University of Aveiro, Aveiro, Portugal

^6^ GreenCoLab - Green Ocean Association, University of Algarve, Faro, Portugal

^7^ Department of Chemistry and Chemical Biology, Rensselaer Polytechnic Institute, Troy, NY, USA

^8^ Centre for Biotechnology and Interdisciplinary Studies, Rensselaer Polytechnic Institute, Troy, NY, USA

^9^ QOPNA & LAQV-REQUIMTE, Department of Chemistry, University of Aveiro, Aveiro, Portugal

^10^ Serviço de Cardiologia, Hospital Santo António, Centro Hospitalar Universitário do Porto, Porto, Portugal

^11^ UMIB, Instituto de Ciências Biomédicas Abel Salazar, University of Porto, Porto, Portugal

^12^ iBiMED – Institute of Biomedicine, School of Health Sciences, University of Aveiro, Aveiro, Portugal

*equally contributing authors

**Corresponding author**

Marisol Gouveia

iBiMED – Institute of Biomedicine, Department of Medical Sciences, University of Aveiro - Building 30, Agras do Crasto - Campus Universitário de Santiago

3810-193 Aveiro, Portugal

E-mail address: [marisolgouveia@ua.pt](mailto:marisolgouveia@ua.pt)

**Supplemental Content**

**eFigure 1 *–*** Coomassie-stained D2D SDS-PAGE gels from pre- (‘baseline’) and post-exercise plasma samples from patients (‘P’) with heart failure with reduced ejection fraction (HFrEF) (gels P1-P5), and spots excised from gels and analysed by mass spectrometry (in red boxes).

**eFigure 2** – Pie charts with the distribution of the relative abundances of the proteins identified in plasma SRA. A. at baseline and B. after the exercise training program.

**eTable 1 -** Human plasma proteins identified by mass spectrometry analysis in HFrEF patients’ plasma SRA spots excised from D2D gels

**eTable 2 -** Immunoglobulins identified by mass spectrometry analysis in HFrEF patients’ plasma SRA spots excised from D2D gels.

**eTable 3 -** Fold change of protein abundance in HFrEF patients’ plasma SRA after exercise training, between each patient sample (paired samples).

**eTable 4 -** Oxidized proteins identified by mass spectrometry analysis in HFrEF patients’ plasma SRA spots excised from D2D gels.

**eTable 5 -** Oxidized immunoglobulins identified by mass spectrometry analysis in HFrEF patients’ plasma SRA spots excised from D2D gels.

**eTable 6 -** List of oxidative modifications identified by mass spectrometry analysis in SRA excised from 2D gels of HFrEF patients’ plasma samples at baseline (includes immunoglobulins).

**eTable 7 -** List of oxidative modifications identified by mass spectrometry analysis in SRA excised from 2D gels of HFrEF patients’ plasma samples after exercise training (includes immunoglobulins).


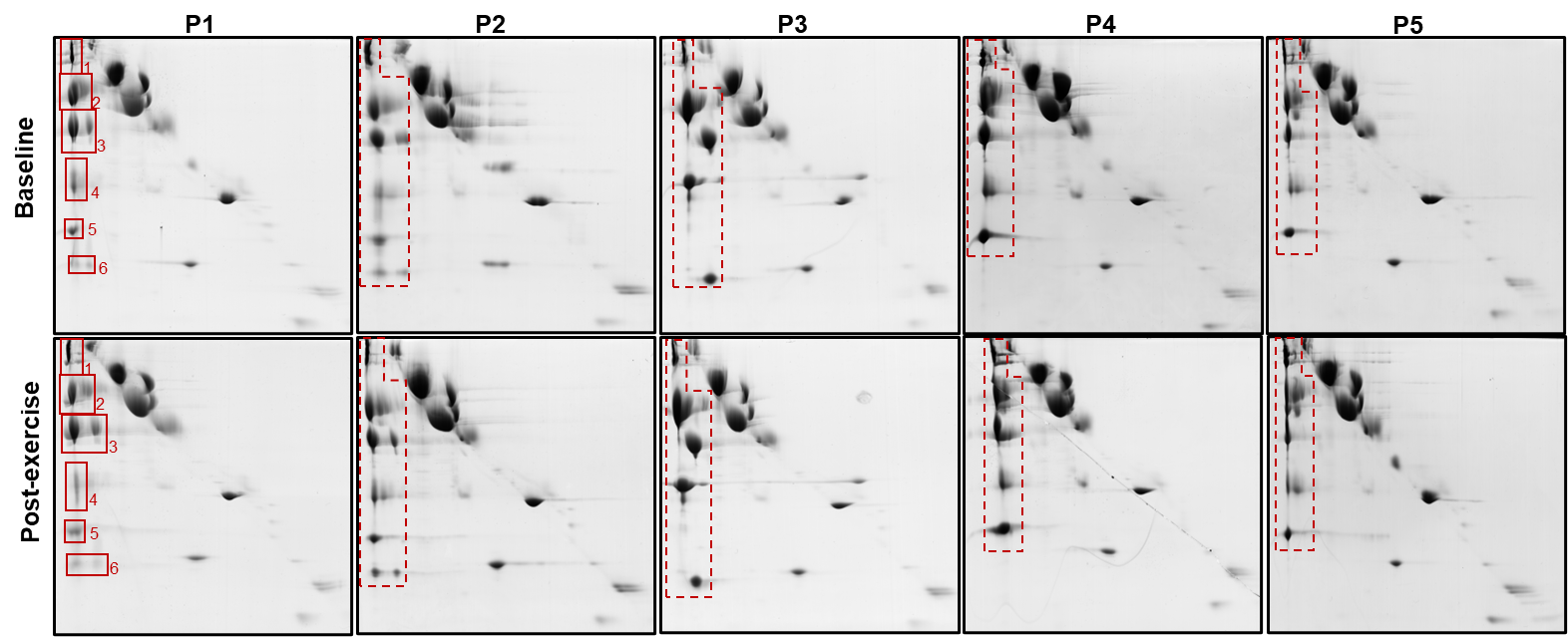


**eFigure 1** *–* Coomassie-stained D2D SDS-PAGE gels from pre- (‘baseline’) and post-exercise plasma samples from patients (‘P’) with heart failure with reduced ejection fraction (HFrEF) (gels P1-P5), and spots excised from gels and analysed by mass spectrometry (in red boxes).


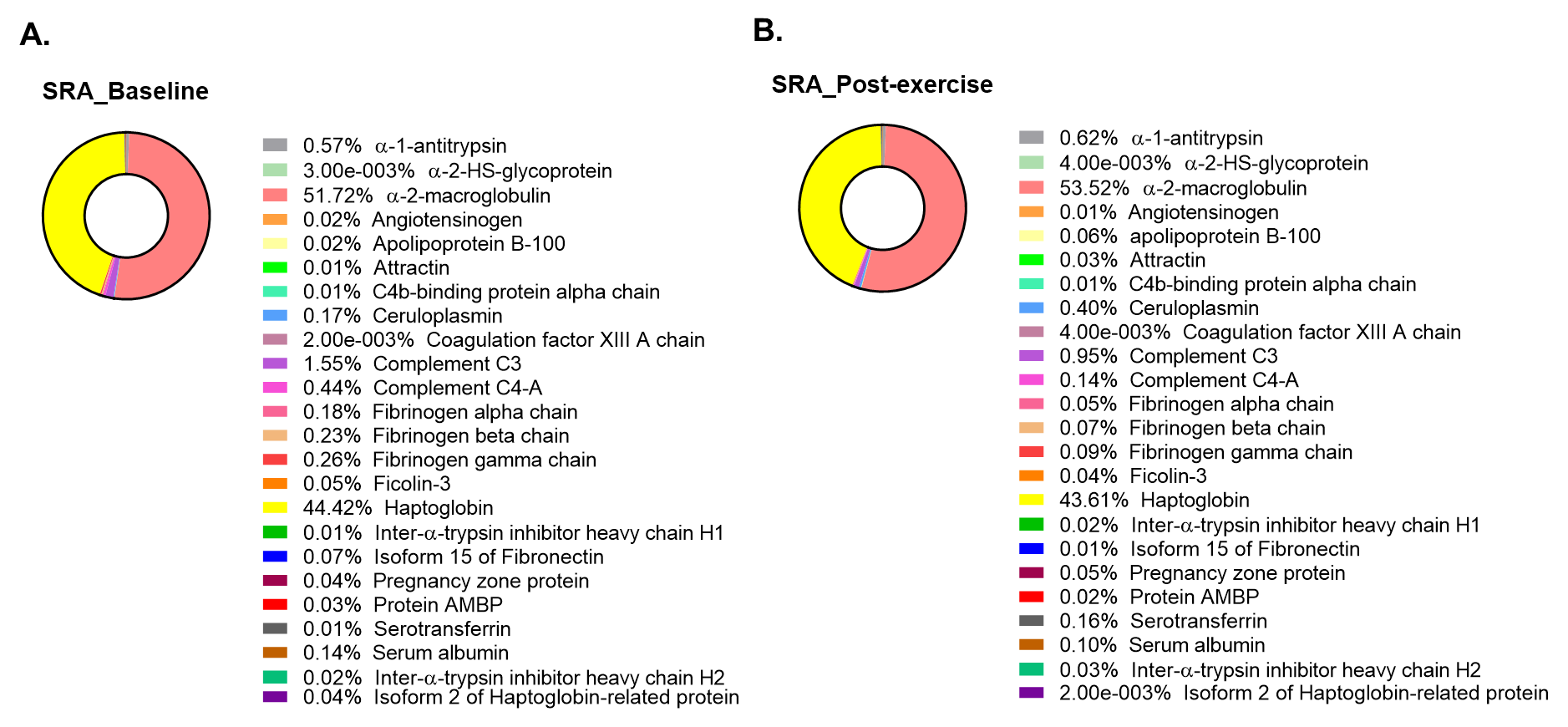


**eFigure 2** – **Pie charts with the distribution of the relative abundances of the proteins identified in plasma SRA**. **A.** at baseline and **B.** after the exercise training program.

**eTable 1** - **Human plasma proteins identified by mass spectrometry analysis in HFrEF patients’ plasma SRA spots excised from D2D gels.** P1 to P5: gels from 5 patients’ plasma samples, assessed at baseline and post-exercise. Grey shadowing, presence of the indicated protein in the indicated SRA gels. (1-X), number of the specific spot(s) where the protein was found (only applicable to P1 samples, where spots were individually analysed; spot numbering as in Supplementary Figure 1). The first 17 proteins (in bold) were identified in at least 8 of the 10 samples analysed. Only proteins that appeared in at least two patients’ samples per timepoint are here presented. Identified immunoglobulins were excluded from this analysis. Abundance: averaged values of the relative abundances (normalized total ion current) of each protein in the 4 patients’ samples where spots were analysed as pools; missing values were replaced by 1/5 of minimum positive values of their corresponding variables. An average post-exercise/baseline fold increase is presented when the corresponding protein appeared in at least two of the P2-P5 samples, per moment of evaluation.

| **Uniprot #** | **Protein name** | **Gene ID** | **Presence in baseline SRA** | | | | | | **Presence in post-exercise SRA** | | | | | | | ***Fold increase*** |
| --- | --- | --- | --- | --- | --- | --- | --- | --- | --- | --- | --- | --- | --- | --- | --- | --- |
|  |  |  | **P1 (spot #)** | **P2** | **P3** | **P4** | **P5** | **Average abundan**  **ce** | **P1 (spot #)** | **P2** | **P3** | **P4** | **P5** | **Average abundance** |  | |
| P01009-1 | **α-1-antitrypsin** | SERPINA1 | 1-3 | 1.6×10^9^ | 4.9×10^9^ | 1.7×10^9^ | 1.1×10^9^ | 2.3×10^9^ | 1-3 | 1.6×10^9^ | 4.8×10^9^ | 1.8×10^9^ | 3.8×10^8^ | 2.1×10^9^ | *0.91* | |
| P01023 | **α-2-macroglobulin** | A2M | 1-6 | 2.3×10^11^ | 1.5×10^11^ | 2.2×10^11^ | 2.5×10^11^ | 2.1×10^11^ | 1-6 | 2.3×10^11^ | 1.8×10^11^ | 1.8×10^11^ | 1.5×10^11^ | 1.9×10^11^ | *0.90* | |
| P01019 | **Angiotensinogen** | AGT | 2 | 1.3×10^8^ | 5.8×10^7^ | 2.1×10^7^ | 6.5×10^7^ | 6.9×10^7^ | 2 | 4.1×10^7^ | 3.0×10^7^ | 4.9×10^7^ | 2.4×10^7^ | 3.6×10^7^ | *0.52* | |
| P00450 | **Ceruloplasmin** | CP | 1-3 | 6.8×10^8^ | 8.2×10^8^ | 8.4×10^8^ | 3.9×10^8^ | 6.8×10^8^ | 1-3 | 2.6×10^8^ | 8.9×10^8^ | 4.0×10^9^ | 3.4×10^8^ | 1.4×10^9^ | *2.06* | |
| P01024 | **Complement C3** | C3 | 1-3 | 2.0×10^9^ | 1.9×10^9^ | 1.3×10^10^ | 8.6×10^9^ | 6.4×10^9^ | 1-3 | 1.8×10^9^ | 2.2×10^9^ | 6.7×10^9^ | 2.4×10^9^ | 3.3×10^9^ | *0.52* | |
| P02671-1 | **Fibrinogen alpha chain** | FGA | 2,3 | 2.1×10^7^ | 2.9×10^7^ | 1.3×10^9^ | 1.6×10^9^ | 7.4×10^8^ |  | 1.5×10^8^ | 1.2×10^7^ | 3.6×10^8^ | 1.6×10^8^ | 1.7×10^8^ | *0.23* | |
| P02675 | **Fibrinogen beta chain** | FGB | 2 | 7.2×10^7^ | 7.9×10^7^ | 1.6×10^9^ | 2.0×10^9^ | 9.4×10^8^ | 2 | 1.2×10^8^ | 7.5×10^7^ | 6.4×10^8^ | 1.7×10^8^ | 2.5×10^8^ | *0.27* | |
| P02679 | **Fibrinogen gamma chain** | FGG | 1-3 | 1.0×10^8^ | 4.9×10^7^ | 2.0×10^9^ | 2.2×10^9^ | 1.1×10^9^ | 2 | 1.4×10^8^ | 1.3×10^8^ | 4.8×10^8^ | 4.7×10^8^ | 3.0×10^8^ | *0.28* | |
| P00738 | **Haptoglobin** | HP | 1-6 | 2.4×10^11^ | 1.9×10^11^ | 1.9×10^11^ | 1.1×10^11^ | 1.8×10^11^ | 1-6 | 1.9×10^11^ | 1.7×10^11^ | 1.8×10^11^ | 6.3×10^10^ | 1.5×10^11^ | *0.83* | |
| P19827-1 | **Inter-alpha-trypsin inhibitor heavy chain H1** | ITIH1 | 1 | 1.1×10^7^ | 2.2×10^6^ | 1.1×10^8^ | 5.3×10^7^ | 4.4×10^7^ | 1 | 1.7×10^7^ | 1.2×10^7^ | 2.2×10^8^ | 4.3×10^7^ | 7.3×10^7^ | *1.66* | |
| P00739-2 | **Isoform 2 of Haptoglobin-related protein** | HPR | 3 | 3.1×10^7^ | 6.5×10^8^ | 6.0×10^6^ | 7.5×10^6^ | 1.7×10^8^ | 3 | 7.4×10^6^ | 1.2×10^7^ | 1.0×10^6^ | 5.0×10^6^ | 6.4×10^6^ | *0.04* | |
| P20742 | **Pregnancy zone protein** | PZP | 1,2 | 3.3×10^8^ | 7.7×10^7^ | 1.2×10^8^ |  | 1.8×10^8^ | 1,2 | 4.1×10^8^ | 3.5×10^7^ | 1.9×10^8^ | 4.5×10^7^ | 1.7×10^8^ | *0.94* | |
| P02760 | **Protein AMBP** | AMBP | 1 | 7.1×10^7^ |  | 1.6×10^8^ | 1.5×10^8^ | 1.3×10^8^ | 1 | 7.2×10^6^ | 4.3×10^7^ | 1.7×10^8^ | 2.7×10^7^ | 6.2×10^7^ | *0.48* | |
| P02787 | **Serotransferrin** | TF | 1,2 | 5.4×10^7^ | 1.7×10^7^ | 3.1×10^7^ | 4.4×10^7^ | 3.7×10^7^ | 1,2 | 1.5×10^7^ | 4.3×10^7^ | 1.6×10^9^ |  | 5.5×10^8^ | *14.86* | |
| P02768-1 | **Serum albumin** | ALB | 1,2 | 5.5×10^8^ | 2.2×10^8^ | 9.6×10^8^ | 6.1×10^8^ | 5.9×10^8^ | 1,2 | 2.2×10^8^ | 3.9×10^8^ | 3.8×10^8^ |  | 3.3×10^8^ | *0.56* | |
| P04003 | **C4b-binding protein alpha chain** | C4BPA | 2 | 1.6×10^7^ | 3.1×10^7^ | 3.1×10^7^ | 4.2×10^7^ | 3.0×10^7^ |  | 2.9×10^7^ | 1.9×10^7^ | 3.8×10^7^ |  | 2.9×10^7^ | *0.97* | |
| O75882-1 | **Attractin** | ATRN | 1,2 | 3.3×10^7^ | 6.8×10^7^ | 1.6×10^7^ | 2.4×10^7^ | 3.5×10^7^ | 1 |  | 1.1×10^8^ | 7.4×10^7^ |  | 9.2×10^7^ | *2.63* | |
| P02765 | α-2-HS-glycoprotein | AHSG | 2 | 1.6×10^7^ | 1.1×10^7^ |  | 5.6×10^6^ | 1.1×10^7^ | 2 | 1.5×10^7^ | 9.9×10^6^ |  |  | 1.2×10^7^ | *1.09* | |
| P04114 | Apolipoprotein B-100 | APOB | 1 |  |  | 2.8×10^7^ | 1.4×10^8^ | 8.4×10^7^ | 1 | 2.8×10^7^ |  | 1.3×10^8^ | 4.4×10^8^ | 2.0×10^8^ | *2.38* | |
| P08697-1 | α-2-antiplasmin | SERPINF2 | 2 | 1.1×10^7^ |  |  |  |  |  | 1.2×10^7^ | 3.4×10^7^ | 1.4×10^7^ |  | 2.0×10^7^ |  | |
| P00488 | Coagulation factor XIII A chain | F13A1 |  | 1.2×10^7^ |  | 3.6×10^6^ |  | 7.8×10^6^ |  | 2.1×10^7^ |  | 8.6×10^6^ |  | 1.5×10^7^ | *1.92* | |
| P0C0L5 | Complement C4-B | C4B |  | 4.0×10^8^ |  | 9.9×10^7^ | 2.2×10^7^ | 1.7×10^8^ |  |  | 1.3×10^7^ | 4.2×10^7^ |  | 2.8×10^7^ | *0.16* | |
| P0C0L4-1 | Complement C4-A | C4A | 2 |  |  | 2.5×10^9^ | 1.1×10^9^ | 1.8×10^9^ | 1,2 |  | 3.9×10^8^ | 8.3×10^8^ | 2.4×10^8^ | 4.9×10^8^ | *0.27* | |
| O75636-1 | Ficolin-3 | FCN3 | 3 |  |  | 1.4×10^8^ | 2.7×10^8^ | 2.1×10^8^ |  |  |  | 8.0×10^7^ | 1.7×10^8^ | 1.3×10^8^ | *0.62* | |
| P19823 | Inter-alpha-trypsin inhibitor heavy chain H2 | ITIH2 |  |  |  | 1.2×10^8^ | 5.2×10^7^ | 8.6×10^7^ |  | 1.2×10^7^ |  | 2.1×10^8^ | 1.4×10^8^ | 1.2×10^8^ | *1.40* | |
| Q06033-1 | Inter-alpha-trypsin inhibitor heavy chain H3 | ITIH3 | 1 |  |  |  | 1.1×10^6^ |  | 1 |  |  | 3.× 10^7^ |  |  |  | |
| P02751-15 | Isoform 15 of Fibronectin | FN1 | 1 |  |  | 1.5×10^8^ | 3.9×10^8^ | 2.7×10^8^ |  | 1.3×10^7^ |  | 2.5×10^7^ | 4.2×10^7^ | 2.7×10^7^ | *0.10* | |
| P48740-2 | Isoform 2 of Mannan-binding lectin serine protease 1 | MASP1 | 2,3 | 6.6×10^6^ |  | 2.6×10^5^ |  | 3.4×10^6^ | 2,3 |  |  | 8.8×10^6^ |  | 8.8×10^6^ |  | |
| P02774-3 | Isoform 3 of Vitamin D-binding protein | GC | 2 | 1.5×10^7^ |  |  |  | 1.5×10^7^ |  | 1.3×10^6^ |  | 8.0×10^6^ |  | 4.6×10^6^ |  | |
| P27169 | Serum paraoxonase/arylesterase 1 | PON1 | 3 | 4.0×10^6^ |  | 2.6×10^5^ |  | 2.1×10^6^ | 3 |  |  | 3.9×10^6^ |  | 3.9×10^6^ |  | |
| P02766 | Transthyretin | TTR | 1,2 | 1.8×10^8^ | 1.2×10^8^ |  |  | 1.5×10^8^ | 1 | 1.1×10^8^ |  |  |  | 1.1×10^8^ |  | |

**eTable 2** - **Immunoglobulins** **identified by mass spectrometry analysis in HFrEF patients’ plasma SRA spots excised from D2D gels.** P1 to P5: gels from 5 patients’ plasma samples, assessed at baseline and post-exercise moments. Grey shadowing, presence of the indicated protein in the SRA spots of the respective gel; (1-X), number of the specific spot(s) where the indicated protein was found (only applicable to P1 samples, where spots were individually analysed; spot numbering as in Supplementary Figure 1). The first 13 protein (in bold) were identified in at least 8 of the 10 samples analysed.

| **Uniprot Accession #** | **Protein Name** | **Presence in baseline SRA** | | | | | **Presence in post-exercise SRA** | | | | |
| --- | --- | --- | --- | --- | --- | --- | --- | --- | --- | --- | --- |
|  |  | **P1**  **(spot #)** | **P2** | **P3** | **P4** | **P5** | **P1**  **(spot #)** | **P2** | **P3** | **P4** | **P5** |
| **P01834** | **Immunoglobulin kappa constant** | 1-4 |  |  |  |  | 1,3-5 |  |  |  |  |
| **P01859** | **Immunoglobulin heavy constant gamma 2** | 2 |  |  |  |  | 2 |  |  |  |  |
| **P01860** | **Immunoglobulin heavy constant gamma 3** | 1-4 |  |  |  |  | 1-4 |  |  |  |  |
| **P0DOX5** | **Immunoglobulin gamma-1 heavy chain** | 1-3 |  |  |  |  | 1,2 |  |  |  |  |
| **P01876** | **Immunoglobulin heavy constant alpha 1** | 1-6 |  |  |  |  | 1-5 |  |  |  |  |
| **P0DOX7** | **Immunoglobulin kappa light chain** | 4 |  |  |  |  | 2,4 |  |  |  |  |
| **P0DOX2** | **Immunoglobulin alpha-2 heavy chain** | 1-3 |  |  |  |  | 1-3 |  |  |  |  |
| **P01591** | **Immunoglobulin J chain** | 4 |  |  |  |  | 4 |  |  |  |  |
| **P01619** | **Immunoglobulin kappa variable 3-20** | 4 |  |  |  |  |  |  |  |  |  |
| **P01861** | **Immunoglobulin heavy constant gamma 4** | 2 |  |  |  |  | 2 |  |  |  |  |
| **P0DOY2** | **Immunoglobulin lambda constant 2** | 5 |  |  |  |  | 1,4 |  |  |  |  |
| **A0A0C4DH38** | **Immunoglobulin heavy variable 5-51** | 2 |  |  |  |  | 2 |  |  |  |  |
| **P01782** | **Immunoglobulin heavy variable 3-9** | 2 |  |  |  |  | 2 |  |  |  |  |
| A0A0A0MRZ8 | Immunoglobulin kappa variable 3D-11 | 4 |  |  |  |  |  |  |  |  |  |
| A0A0B4J1Y9 | Immunoglobulin heavy variable 3-72 | 2 |  |  |  |  |  |  |  |  |  |
| A0A0C4DH31 | Immunoglobulin heavy variable 1-18 | 2 |  |  |  |  | 2 |  |  |  |  |
| A0A0C4DH68 | Immunoglobulin kappa variable 2-24 | 4 |  |  |  |  | 4 |  |  |  |  |
| P01624 | Immunoglobulin kappa variable 3-15 | 4 |  |  |  |  | 4 |  |  |  |  |
| P01700 | Immunoglobulin lambda variable 1-47 | 4 |  |  |  |  |  |  |  |  |  |
| P01701 | Immunoglobulin lambda variable 1-51 |  |  |  |  |  | 4 |  |  |  |  |
| P01780 | Immunoglobulin heavy variable 3-7 | 2 |  |  |  |  | 2 |  |  |  |  |
| P01824 | Immunoglobulin heavy variable 4-39 |  |  |  |  |  |  |  |  |  |  |
| P01871-2 | Isoform 2 of Immunoglobulin heavy constant mu | 2 |  |  |  |  | 2 |  |  |  |  |
| P04433 | Immunoglobulin kappa variable 3-11 |  |  |  |  |  | 4 |  |  |  |  |
| P06310 | Immunoglobulin kappa variable 2-30 |  |  |  |  |  |  |  |  |  |  |
| P06312 | Immunoglobulin kappa variable 4-1 | 4 |  |  |  |  | 4 |  |  |  |  |
| P0DOX3 | Immunoglobulin delta heavy chain |  |  |  |  |  | 2 |  |  |  |  |
| P80748 | Immunoglobulin lambda variable 3-21 |  |  |  |  |  |  |  |  |  |  |

**eTable 3** - **Fold change of protein abundance in HFrEF patients’ plasma SRA after exercise training,** between each patient sample (paired samples). Evaluation timepoints: baseline and post-exercise. Only SRA proteins with at least two paired samples were considered for this analysis.

| **Uniprot #** | **Protein name** | **Presence in baseline SRA** | | | | **Presence in post-exercise SRA** | | | | **Fold change (log2 ratio post- exercise/baseline)** | | | |
| --- | --- | --- | --- | --- | --- | --- | --- | --- | --- | --- | --- | --- | --- |
|  |  | **P2** | **P3** | **P4** | **P5** | **P2** | **P3** | **P4** | **P5** | **P2** | **P3** | **P4** | **P5** |
| P01009-1 | Alpha-1-antitrypsin | 1.6×10^9^ | 4.9×10^9^ | 1.7×10^9^ | 1.1×10^9^ | 1.6×10^9^ | 4.8×10^9^ | 1.8×10^9^ | 3.8×10^8^ | 0.00 | -0.03 | 0.08 | -1.53 |
| P01023 | Alpha-2-macroglobulin | 2.30×10^11^ | 1.5×10^11^ | 2.2×10^11^ | 2.5×10^11^ | 2.3×10^11^ | 1.8×10^11^ | 1.8×10^11^ | 1.5×10^11^ | 0.00 | 0.26 | -0.29 | -0.74 |
| P01019 | Angiotensinogen | 1.3×10^8^ | 5.8×10^7^ | 2.1×10^7^ | 6.5×10^7^ | 4.1×10^7^ | 3.0×10^7^ | 4.9×10^7^ | 2.4×10^7^ | -1.66 | -0.95 | 1.22 | -1.44 |
| P00450 | Ceruloplasmin | 6.8×10^8^ | 8.2×10^8^ | 8.4×10^8^ | 3.9×10^8^ | 2.6×10^8^ | 8.9×10^8^ | 4.0×10^9^ | 3.4×10^8^ | -1.39 | 0.12 | 2.25 | -0.20 |
| P01024 | Complement C3 | 2.0×10^9^ | 1.9×10^9^ | 1.3×10^10^ | 8.6×10^9^ | 1.8×10^9^ | 2.2×10^9^ | 6.7×10^9^ | 2.4×10^9^ | -0.15 | 0.21 | -0.96 | -1.84 |
| P02671-1 | Fibrinogen alpha chain | 2.1×10^7^ | 2.9×10^7^ | 1.3×10^9^ | 1.6×10^9^ | 1.5×10^8^ | 1.2×10^7^ | 3.6×10^8^ | 1.6×10^8^ | 2.84 | -1.27 | -1.85 | -3.32 |
| P02675 | Fibrinogen beta chain | 7.2×10^7^ | 7.9×10^7^ | 1.6×10^9^ | 2.0×10^9^ | 1.2×10^8^ | 7.5×10^7^ | 6.4×10^8^ | 1.7×10^8^ | 0.74 | -0.07 | -1.32 | -3.56 |
| P02679 | Fibrinogen gamma chain | 1.0×10^8^ | 4.9×10^7^ | 2.0×10^9^ | 2.2×10^9^ | 1.4×10^8^ | 1.3×10^8^ | 4.8×10^8^ | 4.7×10^8^ | 0.49 | 1.41 | -2.06 | -2.23 |
| P00738 | Haptoglobin | 2.4×10^11^ | 1.9×10^11^ | 1.9×10^11^ | 1.1×10^11^ | 1.9×10^11^ | 1.7×10^11^ | 1.8×10^11^ | 6.3×10^10^ | -0.34 | -0.16 | -0.08 | -0.80 |
| P19827-1 | Inter-alpha-trypsin inhibitor heavy chain H1 | 1.1×10^7^ | 2.2×10^6^ | 1.1×10^8^ | 5.3×10^7^ | 1.7×10^7^ | 1.2×10^7^ | 2.2×10^8^ | 4.3×10^7^ | 0.63 | 2.45 | 1.00 | -0.30 |
| P00739-2 | Isoform 2 of Haptoglobin-related protein | 3.1×10^7^ | 6.5×10^8^ | 6.0×10^6^ | 7.5×10^6^ | 7.4×10^6^ | 1.2×10^7^ | 1.0×10^6^ | 5.0×10^6^ | -2.07 | -5.76 | -2.58 | -0.58 |
| P20742 | Pregnancy zone protein | 3.3×10^8^ | 7.7×10^7^ | 1.2×10^8^ |  | 4.1×10^8^ | 3.5×10^7^ | 1.9×10^8^ | 4.5×10^7^ | 0.31 | -1.14 | 0.66 |  |
| P02760 | Protein AMBP | 7.1×10^7^ |  | 1.6×10^8^ | 1.5×10^8^ | 7.2×10^6^ | 4.3×10^7^ | 1.7×10^8^ | 2.7×10^7^ | -3.30 |  | 0.09 | -2.47 |
| P02787 | Serotransferrin | 5.4×10^7^ | 1.7×10^7^ | 3.1×10^7^ | 4.4×10^7^ | 1.5×10^7^ | 4.3×10^7^ | 1.6×10^9^ |  | -1.85 | 1.34 | 5.69 |  |
| P02768-1 | Serum albumin | 5.5×10^8^ | 2.2×10^8^ | 9.6×10^8^ | 6.1×10^8^ | 2.2×10^8^ | 3.9×10^8^ | 3.8×10^8^ |  | -1.32 | 0.83 | -1.34 |  |
| P04003 | C4b-binding protein alpha chain | 1.6×10^7^ | 3.1×10^7^ | 3.1×10^7^ | 4.2×10^7^ | 2.9×10^7^ | 1.9×10^7^ | 3.8×10^7^ |  | 0.86 | -0.71 | 0.29 |  |
| O75882-1 | Attractin | 3.3×10^7^ | 6.8×10^7^ | 1.6×10^7^ | 2.4×10^7^ |  | 1.1×10^8^ | 7.4×10^7^ |  |  | 0.69 | 2.21 |  |
| P02765 | Alpha-2-HS-glycoprotein | 1.6×10^7^ | 1.10×10^7^ |  | 5.6×10^6^ | 1.5×10^7^ | 9.9×10^6^ |  |  | -0.09 | -0.15 |  |  |
| P04114 | apolipoprotein B-100 |  |  | 2.8×10^7^ | 1.4×10^8^ | 2.8×10^7^ |  | 1.3×10^8^ | 4.4×10^8^ |  |  | 2.22 | 1.65 |
| P00488 | Coagulation factor XIII A chain | 1.2×10^7^ |  | 3.6×10^6^ |  | 2.1×10^7^ |  | 8.6×10^6^ |  | 0.81 |  | 1.26 |  |
| P0C0L4-1 | Complement C4-A |  |  | 2.5×10^9^ | 1.1×10^9^ |  | 3.9×10^8^ | 8.3×10^8^ | 2.4×10^8^ |  |  | -1.59 | -2.20 |
| O75636-1 | Ficolin-3 |  |  | 1.4×10^8^ | 2.7×10^8^ |  |  | 8.0×10^7^ | 1.7×10^8^ |  |  | -0.81 | -0.67 |
| P19823 | Inter-alpha-trypsin inhibitor heavy chain H2 |  |  | 1.2×10^8^ | 5.2×10^7^ | 1.2×10^7^ |  | 2.1×10^8^ | 1.4×10^8^ |  |  | 0.81 | 1.43 |
| P02751-15 | Isoform 15 of Fibronectin |  |  | 1.5×10^8^ | 3.9×10^8^ | 1.3×10^7^ |  | 2.5×10^7^ | 4.2×10^7^ |  |  | -2.58 | -3.22 |

**eTable 4** - **Oxidized proteins identified by mass spectrometry analysis in HFrEF patients’ plasma SRA spots excised from D2D gels.** P1 to P5: gels from 5 patients’ plasma samples, assessed at baseline and post-exercise moments. Grey shadowing, presence of the indicated protein in the SRA spots of the respective gel; (1-X), number of the specific spot(s) where the indicated protein was found (only applicable to P1 samples, where spots were individually analysed; spot numbering as in Supplementary Figure 1). The first 5 proteins (in bold) were identified in at least 8 of the 10 samples analysed.

| **Uniprot Accession #** | **Protein Name** | **Presence in baseline SRA** | | | | | **Presence in post-exercise SRA** | | | | |
| --- | --- | --- | --- | --- | --- | --- | --- | --- | --- | --- | --- |
|  |  | **P1**  **(spots #)** | **P2** | **P3** | **P4** | **P5** | **P1**  **(spots #)** | **P2** | **P3** | **P4** | **P5** |
| **P01023** | **α-2-macroglobulin** | 1-4 |  |  |  |  | 1-3 |  |  |  |  |
| **P01024** | **Complement C3** | 1,2 |  |  |  |  | 1,2 |  |  |  |  |
| **P00738** | **Haptoglobin** | 1-6 |  |  |  |  | 1-6 |  |  |  |  |
| **P00739-2** | **Isoform 2 of Haptoglobin-related protein** | 3 |  |  |  |  | 3 |  |  |  |  |
| **P20742** | **Pregnancy zone protein** | 1,2 |  |  |  |  | 1,2 |  |  |  |  |
| P02768-1 | Serum albumin |  |  |  |  |  |  |  |  |  |  |
| P00450 | Ceruloplasmin | 1 |  |  |  |  | 1 |  |  |  |  |
| P02787 | Serotransferrin | 1 |  |  |  |  |  |  |  |  |  |
| P02760 | Protein AMBP | 1 |  |  |  |  |  |  |  |  |  |
| P02671-1 | Fibrinogen alpha chain |  |  |  |  |  |  |  |  |  |  |
| P02675 | Fibrinogen beta chain | 2 |  |  |  |  |  |  |  |  |  |
| P02679 | Fibrinogen gamma chain | 2 |  |  |  |  |  |  |  |  |  |
| O75636-1 | Ficolin-3 | 3 |  |  |  |  |  |  |  |  |  |

**eTable 5** - **Oxidized immunoglobulins identified by mass spectrometry analysis in HFrEF patients’ plasma SRA spots excised from D2D gels.** P1 to P5: gels from 5 patients’ plasma samples, assessed at baseline and post-exercise moments. Grey shadowing, presence of the indicated protein in the SRA spots of the respective gel; (1-X), number of the specific spot(s) where the indicated protein was found (only applicable to P1 samples, where spots were individually analysed; spot numbering as in Supplementary Figure 1). The first 9 protein (in bold) were identified in at least 8 of the 10 samples analysed.

| **Uniprot Accession #** | **Protein Name** | **Presence in baseline SRA** | | | | | **Presence in post-exercise SRA** | | | | |
| --- | --- | --- | --- | --- | --- | --- | --- | --- | --- | --- | --- |
|  |  | **P1**  **(spots #)** | **P2** | **P3** | **P4** | **P5** | **P1**  **(spots#)** | **P2** | **P3** | **P4** | **P5** |
| **P0DOX2** | **Immunoglobulin alpha-2 heavy chain** | 1,2 |  |  |  |  | 1-3 |  |  |  |  |
| **P0DOX5** | **Immunoglobulin gamma-1 heavy chain** | 2 |  |  |  |  | 2 |  |  |  |  |
| **P01876** | **Immunoglobulin heavy constant alpha 1** | 1-5 |  |  |  |  | 2,4 |  |  |  |  |
| **P01859** | **Immunoglobulin heavy constant gamma 2** | 2 |  |  |  |  | 2 |  |  |  |  |
| **P01860** | **Immunoglobulin heavy constant gamma 3** | 1-3 |  |  |  |  | 1,2 |  |  |  |  |
| **P01834** | **Immunoglobulin kappa constant** | 4 |  |  |  |  | 4 |  |  |  |  |
| **P0DOX7** | **Immunoglobulin kappa light chain** | 4 |  |  |  |  | 4 |  |  |  |  |
| **P0DOY2** | **Immunoglobulin lambda constant 2** | 4 |  |  |  |  | 1,4 |  |  |  |  |
| **P01782** | **Immunoglobulin heavy variable 3-9** | 2 |  |  |  |  | 2 |  |  |  |  |
| P80748 | Immunoglobulin lambda variable 3-21 |  |  |  |  |  |  |  |  |  |  |

**eTable 6** **- List of oxidative modifications identified by mass spectrometry analysis in SRA excised from 2D gels of HFrEF patients’ plasma samples at baseline (includes immunoglobulins).**

| **Uniprot Accession** | **Protein Name** | **Gel ID** | **Modifications** |
| --- | --- | --- | --- |
| P01023 | α-2-macroglobulin | P2 | Oxidation [K123; N124; R147; D152; N154; P157; N159; D274; D277; N295; Y301; Y316; K319; Y432; Y434; P463; P469; Y480; N483; D519; K521; P529; Y543; N562; N566; K567; D569; P574; K608; P609; D610; P657; N662; K664; D665; Y667; D672; K676; P690; Y695; P845; N851; P902; P907; K912; N917; P922; Y1021; D1022; Y1025; N1035; N1038; K1133; P1202; Y1211; Y1216; P1221; Y1312; K1315; N1377; Y1452; Y1453; D1456]; Dioxidation [M101; R147; M151; P157; Y272; Y316; M318; K319; Y432; Y434; P463; M464; P469; Y480; M500; M520; K521; K567; P574; M688; P690; Y695; M697; M713; M798; Y1021; Y1025; K1133; M1314; K1315; M1385; Y1452; Y1453] |
|  |  | P3 | Oxidation [K123; N124; D126; R147; D152; N154; P157; N159; N295; Y316; K319; P430; Y432; Y434; P463; P469; Y480; N483; K502; D519; K521; Y543; Y558; D559; N562; N566; K567; D569; P574; K608; P609; D610; D638; N639; D641; N651; Y655; P657; N662; K664; D665; Y667; D672; K676; P690; P726; D775; P907; K912; N917; P922; N1038; K1133; Y1211; Y1216; Y1312; K1315; P1340; D1456]; Dioxidation [M101; R147; M151; P157; P218; Y432; Y434; Y480; M500; K502; K567; Y655; P657; M666; Y667; M673; K676; M688; P690; P726; M798; P922; Y1025; K1133; M1314; K1315; M1385] |
|  |  | P1  (spot 1) | Oxidation [D76; N81; D82; N97; K123; N124; D126; R147; D152; N154; P157; N159; P163; P198; P218; K271; Y272; D274; D277; D282; N295; Y301; K314; Y316; K319; K382; P430; Y432; Y434; Y449; P463; P469; Y480; N483; K502; K516; D519; K521; P529; K531; Y543; N562; N566; K567; D569; P574; K608; P633; N635; D636; D638; N639; D641; N644; R645; N647; Y649; N651; Y655; P657; N662; K664; D665; Y667; D672; P690; Y695; Y708; R715; P726; K733; Y734; P736; D742; N747; P791; N851; K896; D897; K901; P902; P907; K912; N917; P922; D1022; N1035; N1038; D1059; K1133; K1147; K1162; R1163; P1202; Y1211; Y1216; P1221; Y1312; K1315; P1334; K1336; P1340; N1377; D1382; K1384]; Dioxidation [M101; M151; P157; P218; K271; Y272; P430; Y432; Y434; Y449; P463; M464; P469; Y480; M500; Y558; K567; P574; P579; R586; M688; P690; M697; Y708; M713; R715; P726; P736; P759; K766; M798; K901; P902; P907; K912; P922; Y1021; Y1025; K1133; Y1145; K1147; P1202; M1208; Y1211; Y1216; Y1312; M1314; K1315; P1334; K1336; M1385] |
|  |  | P1  (spot 2) | Oxidation [D152; N154; P430; Y432; Y434; Y449; P463; Y480; N483; N1038; D1059; K1133; R1163; P1192; Y1197; P1199; P1202; Y1211; Y1216; P1221; K1315; P1340; N1377; Y1452; Y1453]; Dioxidation [K1133; P1202; M1208; Y1211; Y1216; M1314; M1385; Y1452; Y1453] |
|  |  | P1  (spot 3) | Oxidation [Y734; P736; D742; N747; N1038; D1059; K1133; P1202; Y1211; Y1216; P1221; K1315]; Dioxidation [R1073; K1133] |
|  |  | P1  (spot 4) | Oxidation [N97; N747; D1059] |
|  |  | P4 | Oxidation [D76; N81; D82; N97; R147; D152; N154; P157; N159; Y272; D274; D277; N295; Y316; K319; P430; Y432; Y434; Y449; P463; P469; Y480; N483; K502; D519; K521; Y543; N562; N566; K567; D569; P574; K608; P609; P633; N635; D638; N639; D641; N644; R645; N647; Y649; N662; K664; D665; Y667; K676; P690; P726; P791; P845; N851; K901; P902; P907; K912; N917; Y1021; D1022; N1035; N1038; K1133; D1139; Y1145; K1147; P1199; P1202; Y1211; Y1216; P1221; N1377]; Dioxidation [M101; R147; M151; P157; P218; Y272; P430; Y432; Y434; Y449; P463; M464; P469; Y480; M500; M688; P690; M697; M713; R715; P726; M798; Y1021; Y1025; R1073; Y1145; K1147; P1202; M1208; Y1211; Y1216; M1314; K1315; M1385] |
|  |  | P5 | Oxidation [N97; K123; N124; D126; R147; D152; N154; P157; N159; P218; K271; Y272; D274; D277; N295; Y316; K319; P430; Y432; Y434; P463; P469; Y480; N483; K502; D519; K521; P529; D533; Y543; Y558; D559; N562; N566; K567; D569; P574; D601; K608; P609; D610; D638; N639; D641; N644; N651; Y655; P657; N662; K664; D665; Y667; D672; K676; P690; Y695; R715; P726; P845; N851; N1009; Y1021; D1022; N1035; N1038; K1133; Y1211; K1315; P1349; D1353; N1377]; Dioxidation [M101; R147; M151; P157; Y272; Y432; Y434; P463; M464; P469; Y480; M500; P536; R539; Y543; Y558; K567; Y655; P657; K664; M666; Y667; M688; P690; Y695; M697; M713; P726; M798; Y1007; Y1021; Y1025; K1133; M1314; K1315; M1385] |
| P00450 | Ceruloplasmin | P3 | Oxidation [D871] |
|  |  | P1 (spot 1) | Oxidation [P151; Y155; Y157; P318; D323; Y325; N330; P331; Y539; P556; N956; K957] |
|  |  | P4 | Oxidation [P556; R830; P831; Y832] |
| P01024 | Complement C3 | P2 | Oxidation [D48; D52; Y363; K365; P366; P369; D371; P986]; Dioxidation [Y363; K365; P366; M368; P369; M373] |
|  |  | P3 | Oxidation [P986; Y1561] |
|  |  | P1 (spot 1) | Oxidation [N1135; N1136; N1137; K1139; D1140] |
|  |  | P1 (spot 2) | Oxidation [D48; N200; Y363; K365; P366; P369; D371] |
|  |  | P4 | Oxidation [D48; D52; Y363; K365; P366; P369; D371; N1135; N1136; N1137; K1139; D1140; Y1602]; Dioxidation [K365; P366; M368; P369; M373; M1407; M1408; M1563] |
|  |  | P5 | Oxidation [D48; D52; N166; Y363; K365; P366; P369; D371; N378; Y385; R386; P986; N1135; N1136; N1137; K1139; D1140; K1155; D1156; Y1348]; Dioxidation [Y363; K365; P366; M368; P369; M373; M1347; Y1348; M1563] |
| P02671-1 | Fibrinogen alpha chain | P4 | Oxidation [K71; K227; P228; N296] |
|  |  | P5 | Oxidation [K71; K227; P228; P530; P535] |
| P02675 | Fibrinogen beta chain | P1 (spot 2) | Oxidation [K247] |
|  |  | P4 | Oxidation [K52; R53; P57; R60; P61; P63; Y255; D271; N273; K458] |
|  |  | P5 | Oxidation [P57; R60; P61; P63; K247; Y255; D271; N273; P337; D455; N469]; Dioxidation [M468] |
| P02679 | Fibrinogen gamma chain | P1 (spot 2) | Oxidation [P102; N103] |
|  |  | P4 | Oxidation [P102; N103; K121; Y122; P164; K166; D167] |
|  |  | P5 | Oxidation [P102; N103; D106; K114; K121; Y122] |
| O75636-1 | Ficolin-3 | P1 (spot 3) | Oxidation [P47; P50; P53; K56; P73; K74; P77; D79; Y107; P118; D122; D124]; Dioxidation [M71; P73; K74; Y107; M123] |
|  |  | P4 | Oxidation [P47; P50; P53; P73; K74; P77; D79; P118; D122]; Dioxidation [K74] |
|  |  | P5 | Oxidation [P50; P53; K56; P73; K74; P77; D79; D122; D124] |
| P00738 | Haptoglobin | P2 | Oxidation [D81; K82; P84; D89; D90; P93; K94; D122; N128; N129; K131; D140; K141; P143; Y224; P264; P268; K270; Y272; Y280; Y298; P302; D305; K345; D349; Y352; D354; D363; D367; Y370; Y386; Y389; D397]; Dioxidation [P84; Y125; K131; P143; P174; M263; P264; P268; Y280; Y298; M300; M343; K345; Y352; Y370] |
|  |  | P3 | Oxidation [K131; D140; K141; P143; P264; P268; K270; Y280; D294; K297; Y298; N335; K345; D349; Y352; D354; D363; D367; Y370; Y386; D397]; Dioxidation [K72; K131; M263; P264; P268; Y280; K297; Y298; M300; R311; M343; K345; Y352; Y370] |
|  |  | P1 (spot 1) | Oxidation [D81; K82; P84; D89; D90; P93; K94] |
|  |  | P1 (spot 2) | Oxidation [D140; K141; P143; Y298; K345]; Dioxidation [M343; K345] |
|  |  | P1 (spot 3) | Oxidation [D216; Y224; K227; K228; P240; N241; Y242; P264; P268; K270; D271; Y272; N289; K291; D294; K297; Y298; P302; D305; D307; P332; N335; K345; Y346; D349; Y352; D354; D363; D367; Y370; Y386; Y389; D397]; Dioxidation [P219; Y224; P240; Y242; M263; P264; P268; Y280; K297; Y298; M300; P302; P332; M343; K345; Y352; Y370; Y386] |
|  |  | P1 (spot 4) | Oxidation [P264; Y346; D349; Y352; D354; D363; D367; Y370]; Dioxidation [Y346; Y352; Y370] |
|  |  | P1 (spot 5) | Oxidation [D63; Y66; N69; D70; K71; K72; D81; K82; P84; D89; D90; P93; K94; P95; P96; D122; Y125; N128; N129; K131; D140; K141; P143; Y352; D354; D363; D367]; Dioxidation [Y66; K71; K72; K82; P84; P93; K94; P95; P96; Y125; K131; K141; P143; Y352] |
|  |  | P1 (spot 6) | Oxidation [Y125; N128; N129; K131; D140; K141; P143; Y346; D349; Y352; D354; D363]; Dioxidation [Y125; K131; Y352; Y370] |
|  |  | P4 | Oxidation [D63; D81; K82; P84; D89; D90; P93; K94; D122; N128; N129; K131; D140; K141; P143; D216; Y224; P264; P268; K270; D271; Y272; Y280; Y298; P332; N335; K345; D349; Y352; D354; D363; D367; Y370; D397]; Dioxidation [Y66; K82; P84; P93; K94; P95; Y125; K131; P143; P219; M263; P264; P268; Y280; M300; P332; M343; K345; Y352; Y370] |
|  |  | P5 | Oxidation [K72; D81; K82; P84; D89; D90; P93; K94; P95; P96; D122; N128; N129; K131; D140; K141; P143; D216; Y224; P264; P268; Y272; Y280; Y298; P302; P332; N335; K345; D349; Y352; D354; D363; D367; Y370; D397]; Dioxidation [K82; P84; P93; K94; P95; P96; Y102; Y125; K131; P219; M263; P264; P268; K270; Y280; M300; P327; P332; M343; K345; Y352; Y370] |
| P00739-2 | Isoform 2 of Haptoglobin-related protein | P2 | Oxidation [D119; K120; P122; Y203; P247; K249; N250; K324; Y325; D328; Y331; D333; D342; D346; Y349; Y365; Y368]; Dioxidation [P122; P153; M242; M322; K324; Y325; Y331; Y349] |
|  |  | P3 | Oxidation [D119; K120; P122; P247; K249; N250; Y331; D333; D342; D346; Y349; Y365]; Dioxidation [K110; M242; P247; K249; Y331; Y349] |
|  |  | P1  (spot 3) | Oxidation [D195; Y203; K206; K207; P247; K249; N250; P311; N314; K324; D328; Y331; D333; D342; D346; Y349; Y365; Y368]; Dioxidation [P198; Y203; M242; P311; M322; K324; Y331; Y349; Y365] |
|  |  | P4 | Oxidation [D101; D119; K120; P122; D195; Y203; N250; N314; Y325; D328; Y331; D333; D342; D346; Y349]; Dioxidation [Y104; P122; P198; M242; P311; K324; Y325; Y331; Y349] |
|  |  | P5 | Oxidation [K110; D119; K120; P122; D195; Y203; P247; K249; N250; Y331; D333; D342; D346]; Dioxidation [P198; Y349] |
| P20742 | Pregnancy zone protein | P2 | Oxidation [N1041; N1044]; Dioxidation [M804; M1391] |
|  |  | P3 | Oxidation [D781; N1044]; Dioxidation [M804; M1391] |
|  |  | P1  (spot 1) | Oxidation [P465; P797; N1041; N1044]; Dioxidation [M804; M1391] |
|  |  | P1  (spot 2) | Oxidation [N1044; N1139; K1142]; Dioxidation [K1142; M1391] |
|  |  | P4 | Oxidation [P797; N1041; N1044]; Dioxidation [M804; M1391] |
|  |  | P5 | Oxidation [D515; K517; K604; N1041; N1044; N1139]; Dioxidation [M804; K1142; M1391] |
| P02760 | Protein AMBP | P2 | Oxidation [P175; D177; D184; R185] |
|  |  | P1 (spot 1) | Oxidation [P175; D177; D184; R185]; Dioxidation [P175; M182] |
|  |  | P4 | Oxidation [D177; D184; R185] |
|  |  | P5 | Oxidation [P175; D177; D184; R185; P190] |
| P02787 | Serotransferrin | P2 | Oxidation [K278; D280] |
|  |  | P1  (Spot 1) | Oxidation [Y333] |
|  |  | P4 | Oxidation [K278; D280] |
|  |  | P5 | Oxidation [K278; D280] |
| P02768-1 | Serum albumin | P2 | Oxidation [P323; R469; P471] |
|  |  | P3 | Oxidation [N319; D320; R469] |
|  |  | P4 | Oxidation [D320; R469; P471] |
|  |  | P5 | Oxidation [N319; D320; P323; D325; R469; P471] |
| P0DOX2 | Immunoglobulin alpha-2 heavy chain | P2 | Oxidation [P14; D89; Y94; P170; Y179; D280; Y285; P292; P297; N299; N326; R329; P330; P336; P337; P338; R365; K409]; Dioxidation [Y285; P297; R329; P330; P336; P337; P338; R365; K409; M416] |
|  |  | P3 | Oxidation [P148; P151; Y179; P188; P292; N320; K323; N326; R329; P330; P336; P337; P338; N345; R365; K377; Y378; D406; K408; K409]; Dioxidation [R329; P330; P336; P337; P338; K377; Y378; K408; K409; M416] |
|  |  | P1  (spot 1) | Oxidation [N345; K409] |
|  |  | P1  (spot 2) | Oxidation [D89; Y94; R97; Y179; P266; P292; N326; R329; P330; P336; P337; P338; N345; R365; K377; Y378; K409]; Dioxidation [R329; P330; P336; P337; P338; R365; K377; Y378; K409; M416] |
|  |  | P4 | Oxidation [P14; D89; D280; Y285; P292; P297; N299; N345; R365; Y378; K408; K409]; Dioxidation [Y285; R365; K408; K409; M416] |
|  |  | P5 | Dioxidation [P14; R19; P170; Y179; R365; K409; M416]; Oxidation [P14; P170; D173; D177; Y179; N345; R365; K409] |
| P0DOX5 | Immunoglobulin gamma-1 heavy chain | P2 | Oxidation [P125; P129; P132]; Dioxidation [P125; P129] |
|  |  | P3 | Oxidation [P397; P398]; Dioxidation [P397; P398] |
|  |  | P1 (spot 2) | Dioxidation [Y80; M83; R87; P88; Y280; P397; P398]; Oxidation [Y80; N82; N84; R87; P88; D90; P229; P230; P232; Y280; D314; P397; P398] |
|  |  | P4 | Dioxidation [P229; P230; P232; P234; Y280; P397; P398]; Oxidation [P229; P232; P234; P259; D267; Y280; P397; P398; D401; D403] |
|  |  | P5 | Oxidation [P229; P230; P232; P234; P259; D267; Y280; D314; D358; N363; P397; P398; D401; N436]; Dioxidation [Y280; K362; P397; P398] |
| P01876 | Immunoglobulin heavy constant alpha 1 | P2 | Oxidation [P10; P18; D19; N21; P33; P36; P55; D58; D62; Y64; P73; P164; P174; P175; R177; D178; Y183; P190; P195; N197; N224; R227; P228; P234; P235; P236; R263; K306; K307; D309]; Dioxidation [P18; P33; P36; P55; Y64; Y183; P190; P195; R227; P228; P234; P235; P236; R263; K307; M314] |
|  |  | P3 | Oxidation [P10; P33; P36; Y64; P73; P164; P174; P175; R177; D178; Y183; P190; P195; N197; Y208; P214; K221; N224; R227; P228; P234; P235; P236; N243; R263; K275; Y276; P285; K306; K307; D309]; Dioxidation [P10; P33; P36; P174; P175; R177; Y183; P190; P195; P214; K221; R227; P228; P234; P235; P236; K275; Y276; K306; K307; M314; P321] |
|  |  | P1 (spot 1) | Dioxidation [Y183; M314]; Oxidation [P18; D19; N21; P33; Y64; N243; K307; D309] |
|  |  | P1  (spot 2) | Oxidation [P10; P18; D19; N21; P33; P36; Y64; P164; R177; D178; Y183; P190; P195; N197; N224; R227; P228; P234; P235; P236; N243; R263; K275; Y276; P285; K307; D309]; Dioxidation [P18; P33; P36; Y183; P190; P195; R227; P228; P234; P235; P236; R263; K275; Y276; K306; K307; M314] |
|  |  | P1  (spot 3) | Oxidation [P10; P18; D19; N21; P33; P36; Y64] |
|  |  | P1  (spot 4) | Oxidation [P10; P18; D19; N21] |
|  |  | P1  (spot 5) | Oxidation [P10; P18; D19; N21; P33; P36; K307; D309] |
|  |  | P4 | Oxidation [P10; P18; D19; N21; P33; P36; P174; P175; R177; D178; Y183; P190; P195; K221; N224; R227; P228; P234; P235; P236; N243; R263; Y276; K307; D309]; Dioxidation [P10; P18; P33; P36; P174; P175; R177; Y183; P190; P195; K221; R227; P228; P234; P235; P236; R263; M314; P321] |
|  |  | P5 | Oxidation [D62; P164; R177; D178; Y183; P190; P195; N197; K200; N243; R263; K307; D309]; Dioxidation [Y64; P164; R177; Y183; P190; P195; R263; M314; P321] |
| P01859 | Immunoglobulin heavy constant gamma 2 | P2 | Oxidation [P232; R234; P274; P275; D278] |
|  |  | P3 | Oxidation [P232; R234; P274; P275] |
|  |  | P1 (spot 2) | Oxidation [P232; R234; P275] |
|  |  | P4 | Oxidation [P232; R234; P274; P275] |
|  |  | P5 | Oxidation [P231; P232; R234; K239; P274; P275; D280; N313] |
| P01860 | Immunoglobulin heavy constant gamma 3 | P2 | Oxidation [D195; P283; R285] |
|  |  | P3 | Oxidation [P283; R285] |
|  |  | P1  (spot 1) | Oxidation [N351] |
|  |  | P1  (spot 2) | Oxidation [P160; P187; Y208; D242; P283; R285; P325; P326; N351]; Dioxidation [Y208; M358] |
|  |  | P1  (spot 3) | Oxidation [P187; D195; R285] |
|  |  | P4 | Oxidation [P283; R285] |
|  |  | P5 | Oxidation [D195; D242; P282; P283; R285; K290] |
| P01782 | Immunoglobulin heavy variable 3-9 | P2 | Oxidation [D109; Y113] |
|  |  | P3 | Oxidation [Y79; D109] |
|  |  | P1 (spot 2) | Oxidation [D49; D50; Y51; N103; K117]; Dioxidation [Y51] |
|  |  | P5 | Oxidation [Y51; D109] |
| P01834 | Immunoglobulin kappa constant | P2 | Dioxidation [K42; K62; Y79; K81; K83; Y85]; Oxidation [P6; P12; P13; D15; K19; K42; D44; N45; N51; D60; K62; D63; D78; Y79; K81; K83; Y85] |
|  |  | P3 | Oxidation [K83; Y85]; Dioxidation [K83; Y85] |
|  |  | P1 (spot 4) | Oxidation [N30; N31; D44; N45; N51; D60; K62; D78; Y79; K81; K83; Y85]; Dioxidation [K42; K83; Y85] |
|  |  | P4 | Oxidation [D44; N45; N51; D60; K62; D63; Y66; K83; Y85]; Dioxidation [K62; Y66; K83; Y85] |
|  |  | P5 | Dioxidation [K42; K83; Y85]; Oxidation [P12; P13; D15; K19; N30; K42; D44; K83; Y85] |
| P0DOX7 | Immunoglobulin kappa light chain | P2 | Dioxidation [K149; K169; Y186; K188; K190; Y192]; Oxidation [K149; D151; N152; N158; D167; K169; D170; D185; Y186; K188; K190; Y192] |
|  |  | P3 | Oxidation [K190; Y192]; Dioxidation [K190; Y192] |
|  |  | P1  (spot 4) | Oxidation [P8; P113; P120; D122; K126; N137; N138; D151; N152; N158; D167; K169; D185; Y186; K188; K190; Y192]; Dioxidation [M4; P8; P119; P120; K149; K190; Y192] |
|  |  | P4 | Oxidation [D1; N30; D151; N152; N158; D167; K169; D170; Y173; K190; Y192]; Dioxidation [K169; Y173; K190; Y192]; Acetyl [N-Term] |
|  |  | P5 | Oxidation [D1; N137; K149; D151; K190; Y192]; Dioxidation [K149; K190; Y192] |
| P0DOY2 | immunoglobulin lambda constant 2 | P2 | Oxidation [D32; Y34; P35; K43; Y71; K80]; Dioxidation [K43; Y66; Y71; K80] |
|  |  | P3 | Oxidation [D32; Y34; P35; K43; Y66; Y71; P76; K80; Y85]; Dioxidation [K43; Y66; Y71; K80; Y85] |
|  |  | P1  (spot 4) | Oxidation [D32; Y34; P35; K43; N63; N64; K65; Y66; Y71; K80]; Dioxidation [K43; K65; Y66; Y71; P76; K80] |
|  |  | P5 | Dioxidation [K43; Y71; P76; K80]; Oxidation [K43; K80] |
| P80748 | Immunoglobulin lambda variable 3-21 | P3 | Dioxidation [Y54; K57; P58; P62]; Oxidation [Y54; K57; P58; P62] |
|  |  | P5 | Dioxidation [Y54; K57; P58; P62]; Oxidation [Y54; K57; P58; P62] |

**eTable 7 - List of oxidative modifications identified by mass spectrometry analysis in SRA excised from 2D gels of HFrEF patients’ plasma samples after exercise training (includes immunoglobulins).**

| **Uniprot Accession** | **Protein Name** | **Gel ID** | **Modifications** |
| --- | --- | --- | --- |
| P01023 | α-2-macroglobulin | P1 (spot1) | Oxidation [D76; N81; D82; N97; K123; N124; D126; R147; D152; N154; P157; N159; P218; K271; Y272; D274; D277; D282; N295; Y301; K314; Y316; K319; P430; Y432; Y434; Y449; P463; P469; Y480; N483; Y496; Y497; K502; D519; K521; P529; Y543; Y558; D559; N562; N566; K567; D569; R586; D601; K608; P609; D610; Y655; P657; N662; K664; D665; Y667; D672; K676; P690; Y695; Y708; R715; P726; K733; Y734; P736; D742; N747; K766; P791; K896; D897; P907; K912; N917; P922; N1038; D1059; D1097; Y1104; K1133; D1139; Y1145; K1147; P1199; P1202; Y1211; Y1216; P1221; Y1312; N1377; D1382; K1384; Y1452; Y1453; D1456]; Dioxidation [M101; M151; K271; Y272; P430; Y432; Y434; P463; M464; P469; Y480; Y496; Y497; M500; Y558; K567; R598; M607; K608; P609; Y655; P657; M688; P690; Y695; M697; Y708; M713; R715; P726; K733; Y734; P736; P759; K766; M798; P832; P838; Y1021; Y1025; K1133; Y1145; K1147; P1202; M1208; Y1211; Y1216; P1221; M1314; K1315; Y1323; M1385; Y1452; Y1453] |
|  |  | P1 (spot 2) | Oxidation [R147; D152; N154; P157; N159; P430; Y432; Y434; P463; P469; D519; K521; D742; N747; N1035; N1038; D1059; K1133; Y1211; Y1216; K1315; N1377; Y1452; Y1453; D1456]; Dioxidation [P736; Y1025; K1133; M1314; K1315; Y1323; M1385; Y1452; Y1453] |
|  |  | P1 (spot 3) | Oxidation [P430; Y432; Y434; Y734; P736; D742; N747; D1059]; Dioxidation [Y434] |
|  |  | P2 | Oxidation [R147; D152; N154; P157; N159; D274; D277; N295; P430; Y432; Y434; Y449; P463; P469; Y480; N483; K502; D519; K521; P529; Y543; N562; N566; K567; D569; P574; R586; K608; P609; D610; D636; D638; N639; D641; P657; N662; K664; D665; Y667; K676; P690; P726; P845; N851; P907; K912; N917; P922; N1038; K1133; D1139; Y1145; K1147; P1199; P1202; Y1211; Y1216; P1221; Y1312; K1315; N1377; Y1452; Y1453; D1456]; Dioxidation [M101; M151; P463; M464; P469; Y480; M500; K567; M688; P690; M697; M713; P726; M798; P838; K841; P907; K912; P922; Y1025; R1073; K1133; Y1145; K1147; Y1152; P1202; M1208; Y1211; Y1216; P1221; M1314; K1315; M1385; Y1452; Y1453] |
|  |  | P3 | Oxidation [N81; D82; K123; N124; R147; D152; N154; P218; D277; D282; P430; Y432; Y434; P463; P469; Y480; N483; K502; D519; K521; P529; K531; Y543; N562; N566; K567; D569; P574; K608; P609; D610; P657; N662; K664; D665; Y667; P690; P726; P791; P838; K841; P845; Y1021; N1038; K1133; P1202; Y1211; Y1216; K1315; N1377]; Dioxidation [M101; R147; M151; P218; Y432; Y434; P463; P469; Y480; M500; M520; K567; P574; M688; P690; M697; P726; M798; Y1021; Y1025; K1133; P1202; M1208; Y1211; Y1216; M1385] |
|  |  | P4 | Oxidation [D76; N81; D82; N97; R147; D152; N154; P157; N159; D274; D277; N295; P430; Y432; Y434; P463; P469; Y480; N483; K502; K516; D519; K521; Y543; N562; N566; K567; D569; K608; P609; Y655; P657; N662; K664; D665; Y667; D672; K676; P690; P726; P902; P907; K912; N917; P922; Y1021; D1022; N1038; K1133; Y1211; Y1216; P1221; K1315; N1377]; Dioxidation [M101; M151; Y432; Y434; P463; M464; P469; Y480; M500; K502; K567; M688; P690; Y695; M697; M713; R715; P726; Y1021; Y1025; K1133; M1314] |
|  |  | P5 | Oxidation [R147; D152; N154; P157; N159; P430; Y432; Y434; P463; P469; Y480; N483; K521; Y543; N562; N566; K567; D569; K608; D638; N639; D641; D665; Y667; K676; P690; D775; P907; K912; N917; P922; K1133; Y1211; Y1216; K1315]; Dioxidation [M151; P157; P463; Y480; M500; M688; P690; M713; M798; K912; K1133; M1385] |
| P00450 | Ceruloplasmin | P1 (spot 1) | Oxidation [P151; Y155; Y157; Y539; P556; K558] |
|  |  | P3 | Oxidation [N946; K947; D948; D949] |
|  |  | P4 | Oxidation [P556; K558; N826; R830] |
| P01024 | Complement C3 | P1 (spot 1) | Oxidation [N1135; N1136; N1137; K1139; D1140; Y1266; Y1348] |
|  |  | P1 (spot 2) | Oxidation [D624; P629; D641] |
|  |  | P2 | Oxidation [D48; D52; K365; P366; P369; D371; P629; K633; D634; Y635; D641; P986] |
|  |  | P3 | Oxidation [D48; D52; Y1348] |
|  |  | P4 | Oxidation [D48; D52; Y363; K365; P366; P369; D371; P986; N1135; N1136; N1137; K1139; D1140]; Dioxidation [Y363; K365; P366; M368; P369; M373; M1347] |
|  |  | P5 | Oxidation [D48; D52] |
| P02671-1 | Fibrinogen alpha chain | P2 | Oxidation [K227; P228] |
|  |  | P4 | Oxidation [P535] |
|  |  | P5 | Oxidation [K71] |
| P02675 | Fibrinogen beta chain | P2 | Oxidation [N314; Y315; P319] |
|  |  | P4 | Oxidation [P57; R60; P61; P63] |
|  |  | P5 | Oxidation [P107] |
| P02679 | Fibrinogen gamma chain | P2 | Oxidation [P102; N103; P164; K166; D167] |
|  |  | P4 | Oxidation [P102; N103] |
|  |  | P5 | Oxidation [P164; K166] |
| O75636-1 | Ficolin-3 | P4 | Oxidation [P50; P53; K56; D122; D124] |
|  |  | P5 | Oxidation [P50; P53; K56] |
| P00738 | Haptoglobin | P1  (spot 1) | Oxidation [Y298; K345; D349; Y352; D354; D363] |
|  |  | P1  (spot 2) | Oxidation [K345]; Dioxidation [M343; K345] |
|  |  | P1  (spot 3) | Oxidation [N184; Y224; K227; K228; P240; N241; Y242; N259; R261; P264; P268; K270; D271; Y272; Y280; D294; K297; Y298; P302; D307; P332; N335; K345; Y346; D349; Y352; D354; D363; D367; Y370; Y386; Y389; D397]; Dioxidation [P219; Y224; M263; P264; P268; K270; Y280; K297; Y298; M300; P302; R311; P332; M343; K345; Y346; Y352; Y370; Y386] |
|  |  | P1  (spot 4) | Oxidation [P264; Y298; P302; K345; D349; Y352; D354; D363; D367; Y370]; Dioxidation [Y352; Y370] |
|  |  | P1  (spot 5) | Oxidation [D63; D81; K82; P84; D89; D90; P93; K94; D122; Y125; N128; N129; K131; N135; K136; D140; K141; P143; D349; Y352; D354]; Dioxidation [Y66; K82; P84; P93; K94; Y125; K131; K141; P143; K151; Y352] |
|  |  | P1  (spot 6) | Oxidation [D140; K141; P143]; Dioxidation [K131] |
|  |  | P2 | Oxidation [D81; K82; P84; D89; D90; P93; K94; P95; P96; D122; Y125; N128; N129; K131; D140; K141; P143; D216; Y224; P264; P268; Y280; Y298; N335; K345; D349; Y352; D354; D363; D367; Y370; D397]; Dioxidation [K82; P84; P93; K94; Y125; K131; P143; P219; M263; P264; P268; Y280; M300; M343; K345; Y352; Y370] |
|  |  | P3 | Oxidation [K131; D140; K141; P143; D216; Y224; P264; P268; Y280; R286; Y298; D307; K345; D349; Y352; D354; D363; D367; Y370; Y386; Y389; D397]; Dioxidation [P219; M263; P264; P268; Y280; R286; M300; M343; K345; Y352; Y370] |
|  |  | P4 | Oxidation [D63; K71; K72; D81; K82; P84; D89; D90; P93; K94; D122; Y125; N128; N129; K131; D140; K141; P143; D216; P264; P268; K270; Y272; Y280; Y298; D307; K345; D349; Y352; D354; D363; D367; Y370; Y386; D397]; Dioxidation [Y66; K82; P84; P93; K94; Y125; K131; P219; M263; P264; P268; M300; M343; K345; Y352; Y370] |
|  |  | P5 | Oxidation [K131; P264; Y298; K345; D349; Y352; D354; D363; D367; Y370; D397]; Dioxidation [M263; P264; Y280; M300; Y352; Y370] |
| P20742 | Pregnancy zone protein | P1  (spot 1) | Oxidation [K604; P605; P797; N1044]; Dioxidation [M804; M1391] |
|  |  | P1  (spot 2) | Oxidation [N1041; N1044; N1139; K1142]; Dioxidation [K1142; M1391] |
|  |  | P2 | Oxidation [D515; K517; K604; P605; N1044]; Dioxidation [M804; M1391] |
|  |  | P3 | Oxidation [P797; N1044; N1139; K1142]; Dioxidation [M804; K1142; M1391] |
|  |  | P4 | Oxidation [N1044] |
|  |  | P5 | Oxidation [K604; D781]; Dioxidation [M804; M1391] |
| P02760 | Protein AMBP | P3 | Oxidation [P175; D177; D184; R185; P190] |
|  |  | P4 | Oxidation [P175; D177; D184; R185; P190] |
| P02787 | Serotransferrin | P3 | Oxidation [K278; D280] |
|  |  | P4 | Oxidation [K278; D280; Y333; P482] |
| P02768-1 | Serum albumin | P2 | Oxidation [R469; P471] |
|  |  | P3 | Oxidation [N319; D320] |
|  |  | P4 | Oxidation [N319; D320; R469] |
| P0DOX2 | Immunoglobulin α-2 heavy chain | P1 (spot 1) | Oxidation [N345; R365] |
|  |  | P1 (spot 2) | Oxidation [D89; Y93; D173; D177; Y179; P266; Y285; P292; P297; N299; P316; N320; K323; N326; R329; P330; P336; P337; P338; N345; R355; R365; K409]; Dioxidation [Y179; P292; P297; P337; P338; R355; R365; K409; M416] |
|  |  | P1 (spot 3) | Oxidation [K409] |
|  |  | P2 | Oxidation [Y179; P188; Y285; P316; N320; K323; N326; R329; P330; P336; P337; P338; N345; R365; Y378; K409]; Dioxidation [P316; K323; R329; P330; R365; M416] |
|  |  | P3 | Oxidation [D89; Y179; Y285; P292; P316; N320; K323; N326; R329; P330; P336; P337; P338; N345; R365; K377; Y378; K408; K409]; Dioxidation [R329; P330; P336; P337; P338; R365; K377; Y378; K408; K409; M416] |
|  |  | P4 | Oxidation [D89; Y94; D98; R103; K107; Y285; P292; N326; N345; R365; K409]; Dioxidation [R103; K107; Y285; P292; P297; R329; P330; P336; R365; K409; M416] |
|  |  | P5 | Oxidation [N326; N345; R355; D361; R365]; Dioxidation [R329; P330; P336; P337; R365] |
| P0DOX5 | Immunoglobulin gamma-1 heavy chain | P1 (spot 2) | Oxidation [D267; D272; P273; K276; N278; Y280; P354; P355; R357; D358]; Dioxidation [P354; P355; R357; K362] |
|  |  | P2 | Oxidation [P397; P398]; Dioxidation [P397; P398] |
|  |  | P3 | Oxidation [N386; P389; N391; N392; Y393; K394; P397; P398]; Dioxidation [P389; Y393; K394; P397; P398] |
|  |  | P4 | Oxidation [D223; K224; P229; P230; P232; P234; P259; D267; P397; P398]; Dioxidation [P397; P398] |
|  |  | P5 | Oxidation [P232; P234; P259; D267; P397; P398]; Dioxidation [P397; P398] |
| P01876 | Immunoglobulin heavy constant α 1 | P1 (spot 1) | Oxidation [P10; P18; D19; N21; P33; P36; D58; D62; Y64; Y183; N243; R263; K307]; Dioxidation [P10; M314] |
|  |  | P1 (spot 2) | Oxidation [P10; P18; D19; N21; P33; P36; Y64; P164; D178; Y183; P190; P195; N197; N224; R227; P228; P234; P235; P236; N243; R253; R263; Y276; R282; P285; D304; K306; K307; D309; D349]; Dioxidation [P18; P33; P36; Y183; P190; P195; P235; P236; R253; R263; Y276; R282; K306; K307; M314] |
|  |  | P1 (spot 3) | Oxidation [K307; D309] |
|  |  | P2 | Oxidation [P10; P18; D19; N21; P33; P36; Y64; P164; P174; P175; R177; D178; Y183; P190; P195; N197; Y208; N224; P228; P234; P235; P236; N243; R263; Y276; D304; K306; K307; D309]; Dioxidation [P174; P175; R177; Y183; P190; P195; R263; K306; K307; M314; P321] |
|  |  | P3 | Oxidation [P18; D19; N21; P33; P36; P55; D58; D62; Y64; P73; P164; P174; P175; R177; D178; Y183; P190; P195; Y208; P209; N224; R227; P228; P234; P235; P236; N243; R263; K275; Y276; D304; K306; K307; D309; P321]; Dioxidation [P33; P36; P164; P174; P175; R177; Y183; P190; P195; R227; P228; P234; P235; P236; R263; K275; Y276; K307; M314; P321] |
|  |  | P4 | Oxidation [P18; D19; N21; P33; P164; P174; P175; R177; D178; Y183; P190; P195; K200; Y208; N224; N243; R263; K306; K307; D309]; Dioxidation [P36; R177; Y183; P190; R227; P228; P234; R263; K306; K307; M314] |
|  |  | P5 | Oxidation [N224; N243; R253; D259; R263]; Dioxidation [P73; Y183; R227; P228; P234; P235; R263; M314] |
| P01859 | Immunoglobulin heavy constant gamma 2 | P1 (spot 2) | Oxidation [P232; R234; K239] |
|  |  | P2 | Oxidation [P232; R234; P274; P275] |
|  |  | P3 | Oxidation [P232; R234; K239; P274; P275; D278; D280] |
|  |  | P4 | Oxidation [P232; R234; P275] |
|  |  | P5 | Oxidation [P107; P108; P110; P112; P232; R234; K239; P275] |
| P01860 | Immunoglobulin heavy constant gamma 3 | P1  (spot 1) | Oxidation [Y303; P325; P326; D329] |
|  |  | P1  (spot 2) | Oxidation [P160; P187; D195; Y208; P283; R285; K290; N351]; Dioxidation [Y208; M358] |
|  |  | P2 | Oxidation [P187; D195; P283; R285] |
|  |  | P3 | Oxidation [P283; R285; K290] |
|  |  | P4 | Oxidation [D151; R158; P283; R285]; Dioxidation [R158] |
|  |  | P5 | Oxidation [P283; R285; K290] |
| P00739-2 | Isoform 2 of Haptoglobin-related protein | P1  (spot 3) | Oxidation [N163; Y203; K206; K207; P311; N314; D328; Y331; D333; D342; D346; Y349; Y365; Y368]; Dioxidation [P198; Y203; Y331; Y349; Y365] |
|  |  | P2 | Oxidation [D119; K120; P122; D195; Y203; D328; Y331; D333; D342; D346; Y349]; Dioxidation [P122; P198; Y331; Y349] |
|  |  | P3 | Oxidation [D119; K120; P122; D195; Y203; D328; Y331; D333; D342; D346; Y349; Y365; Y368]; Dioxidation [P198; Y331; Y349] |
|  |  | P4 | Oxidation [D101; K109; K110; D119; K120; P122; D195; P247; K324; Y331; D333; D342; D346; Y349; Y365]; Dioxidation [Y104; P198; P243; P247; Y331; Y349] |
|  |  | P5 | Dioxidation [Y349]; Oxidation [Y331; D342; D346; Y349] |
| P01782 | Immunoglobulin heavy variable 3-9 | P1  (spot 2) | Oxidation [N103] |
|  |  | P4 | Oxidation [D109] |
| P01834 | Immunoglobulin kappa constant | P1  (spot 4) | Oxidation [P6; P12; P13; D15; K19; N30; N31; K83; Y85]; Dioxidation [K83; Y85] |
|  |  | P2 | Dioxidation [K62; Y66]; Oxidation [N51; D60; K62; D63; Y66; Y85] |
|  |  | P3 | Oxidation [N51; D60; K62; D63; Y66; K83; Y85]; Dioxidation [K42; K62; Y66; K83; Y85] |
|  |  | P4 | Dioxidation [K83; Y85]; Oxidation [P6; P12; P13; D15; K19; N30; N31; K83] |
|  |  | P5 | Oxidation [N30] |
| P0DOX7 | Immunoglobulin kappa light chain | P1  (spot 4) | Oxidation [D1; P8; N137; N138; K190; Y192]; Acetyl [N-Term]; Dioxidation [K190; Y192] |
|  |  | P2 | Oxidation [D1; N158; D167; K169; D170; Y173; Y192]; Dioxidation [K169; Y173] |
|  |  | P3 | Oxidation [N158; D167; K169; D170; Y173; K190; Y192]; Dioxidation [K149; K169; Y173; K190; Y192] |
|  |  | P4 | Oxidation [N30; N137; N138; K190]; Dioxidation [K190; Y192]; Acetyl [N-Term] |
|  |  | P5 | Oxidation [D1; N137]; Acetyl [N-Term] |
| P0DOY2 | Immunoglobulin lambda constant 2 | P1  (spot 1) | Dioxidation [K43]; Oxidation [K43] |
|  |  | P1  (spot 4) | Oxidation [D32; Y34; P35; K43; Y66; Y71; P76; K80; Y85]; Dioxidation [K43; Y66; Y71; P76; K80; Y85] |
|  |  | P2 | Dioxidation [K43; Y71; K80]; Oxidation [D32; Y34; K43; Y66; K80] |
|  |  | P3 | Oxidation [D32; Y34; K43; Y66; Y71; P76; K80; Y85]; Dioxidation [K43; Y66; Y71; K80; Y85] |
|  |  | P4 | Oxidation [D32; Y34; K43; Y66; Y71; P76; K80]; Dioxidation [K43; Y66; Y71] |
| P80748 | Immunoglobulin lambda variable 3-21 | P3 | Dioxidation [Y54; K57; P58; P62]; Oxidation [Y54; K57; P58; P62] |
|  |  | P4 | Oxidation [Y54; P62] |
